# Plant conservation in the age of genome editing: opportunities and challenges

**DOI:** 10.1186/s13059-024-03399-0

**Published:** 2024-10-24

**Authors:** Kangquan Yin, Mi Yoon Chung, Bo Lan, Fang K. Du, Myong Gi Chung

**Affiliations:** 1 School of Grassland Science, Beijing Forestry University, Beijing, 100083 China; 2 Department of Biological Sciences, Chungnam National University, Daejeon, 34134 South Korea; 3 School of Ecology and Nature Conservation, Beijing Forestry University, Beijing, 100083 China; 4 Division of Life Science, Gyeongsang National University, Jinju, 52828 South Korea

**Keywords:** CRISPR, Climate change, Genomics, Deleterious mutation, Conservation, Genetic diversity

## Abstract

Numerous plant taxa are threatened by habitat destruction or overexploitation. To overcome these threats, new methods are urgently needed for rescuing threatened and endangered plant species. Here, we review the genetic consequences of threats to species populations. We highlight potential advantages of genome editing for mitigating negative effects caused by new pathogens and pests or climate change where other approaches have failed. We propose solutions to protect threatened plants using genome editing technology unless absolutely necessary. We further discuss the challenges associated with genome editing in plant conservation to mitigate the decline of plant diversity.

**Supplementary Information:**

The online version contains supplementary material available at 10.1186/s13059-024-03399-0.

## Background

Plants play central roles in ecosystem dynamics as they provide essential resources for both consumers and decomposers. However, recent studies have confirmed that the sixth extinction event began approximately 100,000 years ago, coinciding with the migration of modern humans to different parts of the world, and is still ongoing [1]. There are unprecedented rates of population decline of many plant species, primarily due to habitat destruction or overexploitation [2]. For example, analyses of seed plant datasets indicate that annual losses of species have been about 500 times higher than the background extinction rate recently [3–5]. Thus, there is an urgent need to conserve or restore many species [6], and for this genetic information about their populations is essential.

Accordingly, in the past three decades, substantial progress has been made in plant conservation genetics and genomics by estimating (putatively) neutral genetic variation (hereafter NGV) in threatened or endangered species using neutral genetic markers (e.g., allozymes, microsatellites [SSRs], or single nucleotide polymorphisms [SNPs]) [7–12]. This can provide valuable indications of species’ demographic history, as well as genetic diversity, and hence appropriate conservation strategies. For example, species may have moderate or high levels of variation, despite small population sizes, because of sharp recent declines [13, 14]. This is believed to apply to many taxa included in China’s Plant Species with Extremely Small Populations program [15]. Most species in the program have very small populations, but in many cases, they have only become small recently and still retain high genetic variation [16]. However, some endangered plant species with small population sizes have extremely low variation, usually attributed to random genetic drift and/or inbreeding or historical events, such as demographic fluctuations due to climate oscillations during the Quaternary [17–21].

The species with low abundance and low genetic variation are of greatest concern because to survive and reproduce plants must cope with environmental changes through adaptive genetic variation (hereafter AGV) [22]. Therefore, maintaining sufficient genetic diversity is essential for species to adapt to changing environments [23–25]. There is also increasing interest in *directional* and *non-directional* responses and their effects on species’ adaptive potential [26], which have been explored in traditional common garden and reciprocal transplant experiments [27, 28]. In addition, genomic information has been used in combination with spatial models recently to analyze probable genetic changes that may result from ecological or local adaptation [29–31]. In particular, interactions between the AGV of species and landscape characteristics have been studied at the genome level to quantify responses to environmental change (i.e., landscape genomics). Recently, genetic offset [32], risks of non-adaptedness (RONA) [33], and genomic vulnerability [34] have also been evaluated to predict climate-driven shifts of genetic compositions within populations [35, 36] and thus, using assisted gene flow and assisted migration, to maximize the local adaptation of focal populations under climate change [37]. Another approach that is being increasingly used in efforts to enhance plants’ adaptation, e.g., to climate change, is “conservation epigenetics,” i.e., the use of epigenetic information on DNA methylation patterns to recommend conservation- or translocation-based “population reinforcement strategies” for target plants [38, 39]. Furthermore, gene expression study (transcriptome analysis) is precious for finding genes and gene networks involved in plants’ local adaptation [29]. Clearly, it is important to understand both NGV- and AGV-based approaches that can be applied in plant conservation or restoration efforts (see the below details in the “Outline of applications of NGV and AGV in plant conservation or restoration” section). This is because knowledge of NGV is essential for understanding many relevant demographic, ecological, and evolutionary processes. Similarly, knowledge of AGV is essential for evaluating plants’ adaptive potential in local environments with changing conditions, e.g., due to global warming, and identifying appropriate conservation units or formulating other appropriate strategies.

Unlike these approaches that can be applied for monitoring and managing existing genomes of species, new genome-based technology that can manipulate genomes is urgently needed to mitigate the negative effects. A decade ago, clustered regularly interspaced short palindromic repeats (CRISPR)-Cas9-mediated site-specific genome engineering was successfully demonstrated in human cells [40–43]. Since then, the approach has blossomed and become the dominant technology for editing genomes of other eukaryotes, including plants [44–46] (see the below details in the “Genome editing tools for plant conservation” section). Recently developed genome editing (hereafter GE, including gene [genome] edited) technology has been widely used to improve traits and investigate gene functions of crops and model plants, mainly because of its ease of design, low cost, and ability to provide transgene-free edits [47–49]. For example, GE has several advantages over conventional mutagenesis methods, like chemical mutagens (e.g., ethyl methanesulfonate [EMS]) or physical irradiation (e.g., fast neutrons), which are more random and less targeted [50]. Multiplex genome editing offers the advantage of manipulating more sites simultaneously, which significantly reduces the time required to generate variants with multiple advantageous mutations. However, the application of GE in plant conservation is still in its infancy despite its ability to generate suitable novel genotypes for challenging conditions [51, 52]. Therefore, GE has a high potential for future applications in conservation and restoration. In this review, we discuss the selection of species, populations, and individuals for GE, the technological basis of GE, propose potential ways to protect threatened plants using GE, discuss the challenges (e.g., technical difficulties, negative impacts on the ecosystem, and regulatory aspects) associated with GE in plant conservation, and provide the strategies for reinforcement of declining wild populations.

### Outline of applications of NGV and AGV in plant conservation or restoration

About 17 applications of population genetics information based on neutral markers (i.e., NGV) are known. These are to (i) estimate NGV within and among populations, (ii) identify conservation units (e.g., management units), (iii) estimate current effective population size, (iv) infer random genetic drift, (v) estimate levels of gene flow, (vi) infer colonization histories of native species and histories of plant invaders, (vii) identify probable glacial refugia, (viii) infer mating systems and parentage, (ix) address issues related to wildlife forensics, (x) estimate levels of inbreeding, (xi) use environmental DNA approaches, (xii) infer human-mediated disturbance, (xiii) identify quantitative trait loci (QTL), (xiv) estimate SNP-based heritability, (xvi) infer historical patterns of dispersal, (xvii) monitor levels of NGV of artificially propagated endangered species, and (xviii) identify clonal and fine-scale genetic structure.

**Fig. 1:**
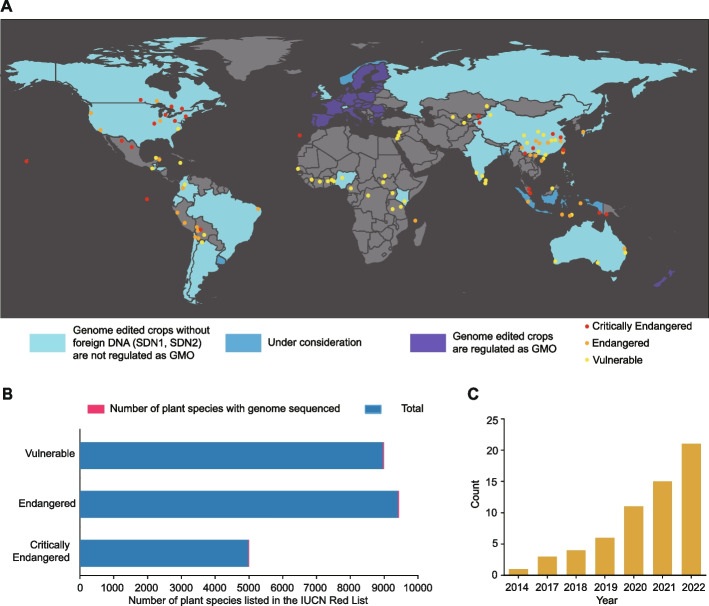
Genome sequencing status of threatened plants.** A** Map of threatened plants with sequenced genomes (red, orange, and yellow dots represent “critically endangered,” “endangered,” and “vulnerable” plants, respectively), and global regulatory status of genome-edited (GE) plants in indicated areas (adapted from [53]). SDN, site-directed nuclease; GMO, genetically modified organism. **B** Genome sequencing status of threatened plants. Total numbers of species (in blue) and numbers with sequenced genomes (in red) in three groups of threatened plants. **C** Increase in numbers of sequenced genomes of threatened plants with time between 2014 and 2022

Similarly, genetic information on AGV has been applied for conservation purposes. These are to (i) estimate AGV within and among populations, (ii) identify conservation units (e.g., adaptive units), (iii) decipher the genetic basis of adaptation to climate change, (iv) predict species range losses under climate change, (v) identify QTL, (vi) identify loci strongly correlated to particular environmental variables, (vii) prevent overestimation of future biodiversity losses, (viii) develop reliable predictions about adaptive potential, (ix) understand genetic mechanisms of inbreeding and outbreeding depression, (x) investigate gene flow associated with adaptive responses, (xi) investigate the balance of gene flow and selection under climate change, (xii) estimate SNP-based heritability, (xiii) compare *Q*_ST_ and *F*_ST_, and (xiv) provide crucial information for assisted migration in trees under climate change. All presented information was modified from Fig. 1 in a previous publication [11].

### Genome editing tools for plant conservation

To date, four types of genetic engineering of plants have been developed: (i) targeted mutagenesis by non-homologous end joining (NHEJ) [47], (ii) precise editing by homology-directed repair (HDR) [54], (iii) base editing (BE) [55], and (iv) prime editing (PE) [56]. These new technologies have the potential to enable rapid and precise manipulation of genes associated with desired traits, and thus greatly assist urgent conservation efforts [12].

Currently, at least six Cas proteins are available for targeted mutagenesis. Cas9 is the most popular CRISPR system adapted for GE in plants. It is quite simple and efficient, requiring Cas9 and a single guide RNA to assemble and then cleave genomic sites after recognition of a targeted DNA sequence followed by a protospacer adjacent motif (PAM). To expand target ranges, Cas9 proteins have been engineered through directed evolution or rational design to obtain variants that recognize different PAMs [57, 58]. In addition, Cas9 orthologs from other bacterial and archaeal organisms have been mined to obtain new tools that recognize different PAMs, such as SaCas9 [59] and FnCas9 [60]. In contrast to Cas9, Cas12a and Cas12b are type V endonucleases that recognize T-rich PAMs and a short CRISPR RNA, which is advantageous for synthesizing and multiplex editing [61, 62]. Moreover, they generate a double-strand break (DSB) with staggered ends distal to the PAM site. CasX requires a guide RNA and trans-activating CRISPR, generating a staggered end with a 10 nt overhang [63]. The Cas proteins mentioned above are assigned to class 2, but members of another class 1 form multimeric complexes that can generate large deletions of up to 100 kb, with a guide RNA [64]. As more Cas proteins are being characterized, the CRISPR toolbox is expanding, and theoretically every site in the genomes of threatened plant species can be targeted.

While NHEJ can efficiently generate mutations in target regions (mostly + 1/ − 1 mutations at the − 3 position upstream of a PAM site), not as precisely as HDR. However, the application of HDR is extremely difficult in higher plants, although DSBs generated by sequence-specific nucleases promote its efficiency [65]. There has been an intense effort to enhance this technology through improvements in donor design, donor availability, and manipulation of DNA repair pathways. Increasing donor availability is a feasible way to improve HDR in plants. For example, the geminivirus replicon has been harnessed to amplify DNA donors in cells to increase HDR efficiency in *Arabidopsis*, tomato, and tobacco [66–68]. Moreover, physical methods involving particle bombardment to deliver dsDNA or ssDNA donors to increase donor amounts have been demonstrated in rice [69, 70].

As some deleterious variants in threatened plant species are point mutations, tools that can edit a single nucleotide are needed [71]. BE is a recently developed breakthrough technology that can introduce point mutations precisely at target sites without the need for DSB and donor DNA [72]. Currently, there are three types of BE tools: cytosine base editors (CBEs), adenine base editors (ABEs), and glycosylase base editors (GBEs). These tools all have similar architecture, including Cas9 nickase and deaminase. The cytidine deaminase of a CBE deaminates cytosine in an exposed non-target DNA strand and generates a U:G mismatch, which is then converted to U:A and finally becomes T:A after replication (resulting in C:G to T:A conversion). This process is promoted by uracil glycosylase inhibition by an inhibitor (UGI) fused to nCas9. Interestingly, GBEs were developed by replacing the UGI with uracil-DNA glycosylase in some CBEs and enabling base transversions (C to A or C to G) [73, 74]. In contrast, ABEs use adenosine deaminase to deaminate adenine (A) into inosine (I) in the non-target strand, without the need to inhibit base excision repair activity, and finally generate A-to-G base conversion [75]. These BEs cannot generate all possible base substitutions, so new BE tools or other technologies are required. PE was developed shortly after BE and can generate a much wider range of mutations of any type, including not only point mutations but also indels, and even complex combinations of mutations [76]. The PE system is composed of three main components: nCas9 (H840A), M‐MLV reverse transcriptase (RT) domain, and pegRNA, which contains a sgRNA, a primer binding sequence (PBS), and a RT template (containing the desired DNA sequence change). RT was fused to nCas9 to generate the prime editor, which is guided to target sequences by the sgRNA. Once nCas9 nicks the PAM-containing non-target strand, PBS will hybridize with the resulting 3′ end which serves as a DNA primer for reverse transcription by the RT domain. Then, the RT domain guides the polymerization of edited DNA from the pegRNA to the target site, resulting in either a 3′ flap with an edit or a 5′ flap without an edit. Flap excision and ligation finally generate heteroduplex DNA with one edited strand and one unedited strand. Through DNA repair or replication that can copy the edit to the complementary strand, the desired edit was permanently installed. This makes PE suitable for all point mutations, insertions, and deletions.

## Determining species, population(s), and individuals to use for genome editing

Under what circumstances is GE applied among endangered plants (included in the IUCN Red List [77]) in comparison with traditional population genetics/genomics and what is the most scientific and efficient way to select target species? When selecting target species, is their demographic, ecological, and biological information well known? Before any GE can commence, there must be a clear justification for the need to apply GE to the target species (e.g., the main purpose of GE and conservation challenges or implications; Table 1). At the same time, researchers should sufficiently justify reasons for species selection by considering the two issues: (i) technical bottlenecks that may arise in introducing GE approaches to the focal species and (ii) the negative effects of GE, which introduce in the “Challenges facing plant conservation using genome editing” section later in this review. By doing so, researchers are likely to be able to define conservation problems more clearly and explore potential solutions more deeply. Currently, most GE research is mainly led by plant biotechnologists, so when selecting a focal species, it would be beneficial to discuss with researchers who major in systematics/taxonomy, population/community ecology, conservation genetics, population genetics (genomics), and bioinformatics. Consultation with managers and practitioners may also be necessary.

**Table 1:** Seven of the whole genome sequenced threatened species successfully implemented genome editing (GE) by *Agrobacterium* or biolistic-mediated delivery, main purpose or motivation, GE tools, and conservation challenges and/or implications

Species	Status^a^	Family	Main purpose of GE	GE tools	Efficiency of GE	Conservation challenge or implication
*Dendrobium officinale*	CR	Orchidaceae	To reduce the lignocellulose content by knocking out genes in the lignocellulose biosynthesis pathway (*C3H*, *C4H*, *4CL*, *CCR*, and *IRX*) [78]	CRISPR/Cas9	10–100^b^	*D. officinale* has been one of the best herbal medicines and food in China and Korea, so wild individuals are rare due to overharvesting. Research is underway on the well-established transformation systems in *D. officinale* and the application of GE to create new varieties. Perhaps, in the face of future abiotic stress, established and secured GE techniques (e.g., knocking out deleterious genes, replacement of high temperature or drought resistance genes) may be needed for the conservation of remaining populations
*Fraxinus nigra*	CR	Oleaceae	To achieve transgene containment, reproductive sterility can be produced by disrupting the *FnAG* gene, which is involved in the development of floral organs [79]	CRISPR/Cas9	2^b^	There has been a great demand for ash trees genetically engineered to be resistant to emerald ash borer. However, widespread acceptance and regulatory approval of transgenic trees has been limited due to concerns about transgene flow and potential impacts on the environment. Reproductive sterility achieved by disrupting flower development is one of several efficient strategies for gene containment in transgenic crops and trees. The conservation implications of this approach would include mitigating local ecosystem disturbances
*Eucalyptus urophylla*	EN	Myrtaceae	To prevent sexual dispersal by pollen and induce male sterility for hybrid breeding by knocking out one involved in meiosis (*EREC8*) and two regulating early stages of pollen development (*ETDF1* and *EHEC3‐like*). Note that the GE species is the hybrid *Eucalyptus grandis* x *E. urophylla* [80]	CRISPR/Cas9	73–100^b^	Eucalyptus commonly spreads beyond exotic forest plantations into wild ecosystems, where it could outcompete native trees, reduce water availability, damage local biodiversity, and disturb local ecosystems. Tools to mitigate spread, such as male and/or complete sterility, may be crucial for ecosystem conservation and sustainability and could protect endangered eucalyptus
*Taxus chinensis*	EN	Taxaceae	To control paclitaxel biosynthesis by knockdown of *PAL* gene [81]	CRISPR-guided DNA methylation	Note that there was a 61% increase of DNA methylation in the 5′-UTR region of *PAL* gene which resulted in approximately a 25-fold increase in paclitaxel accumulation^c^	Manipulating phenylpropanoid biosynthesis in *Taxus* species using GE techniques is highly effective in increasing taxane production. If problems with biometabolism may arise in the future probably caused by abiotic factors, newer approaches based on these techniques could be used for in situ and ex situ conservation of this endangered species
*Vanilla planifolia*	EN	Orchidaceae	To enable targeted domestication of vanilla and to demonstrate it by knocking out the *PDS* gene that is vital to plastid development and chlorophyll biosynthesis [82]	CRISPR/Cas9	92^b^	Successful GE of the *V. planifolia PDS* gene may pave the way for genetic improvements of domestication traits (e.g., dehiscence of fruits, aroma, disease resistance, and stress tolerance). Interested researchers could manipulate from dry and dehiscent nonaromatic fruits to aromatic indehiscent ones using GE technology. Recent studies have shown that wild indehiscent Neotropical *Vanilla* fruits are dispersed by mammals [83]. From a conservation perspective, researchers may use GE techniques if populations of these species have dehiscent fruits due to environmental factors. [This approach may require caution because, in indehiscent Neotropical *Vanilla*, individuals with dehiscent fruits may have lower fitness than indehiscent individuals]
*Dionaea muscipula*	VU	Droseraceae	To test whether mechanosensitive ion channel genes control the Venus flytrap’s hunting behavior by knocking out hair-expressed *MscS-like* genes (*FLYC1* and *FLYC2*) [84]	CRISPR/Cas9	Not available	How does the Venus flytrap detect animal prey for nutrients? How will the mechanosensory ion channels respond due to probable environmental change? As for conservation implications, if some carnivorous plants have problems with their mechanosensory ion channels due to probable environmental factors and their leaves do not function properly, the CRISPR-Cas9 system can be used to enable normal responses
*Gossypium hirsutum*	VU	Malvaceae	To facilitate cotton molecular breeding by precisely targeted insertion of additional trait (herbicide tolerance) genes (*hppd*, *epsps*) using re-engineered meganuclease; to improve the abiotic stress tolerance in cotton by knocking out eleven genes (*GhPDS*, *GhCLA1*, *GhEF1*, *GhVP*, *GhARG*, *GhMYB25-like A*, *GhMYB25-like D*, and *GoPGF*); to promote cotton hybrid breeding by knocking out potential male sterility-related genes to establish gene editing systems using CRISPR/Cas9; and to demonstrate them by knocking out above eleven genes [85–87]	Homologous recombination-mediated targeted insertion (gene stacking) using COT-5/6 meganuclease; CRISPR/Cas9; CRISPR/LbCpf1; CRISPR/Cas12b	48–100^b^	Wild species of *Gossypium* serve as a reservoir of genetic variation for resistance to pests and diseases, as well as tolerance to abiotic stresses [88]. Using genome information in the useful agronomic traits of these species, to develop agronomic and quality traits for cultivated cotton, multi-trait genes are developed for introgressions such as herbicide tolerance, pest (e.g., nematodes, aphids, and spider mites) and disease (e.g., leaf curl virus, *Fusarium* and *Verticillium* wilts) resistance, abiotic stress (e.g., mild frost, drought, and salinity) tolerance, and yield enhancement. These GE techniques could be applied to conserve and preserve (e.g., buffering population decline due to climate change) wild populations of *G. hirsutum*, growing wild in its native Central America, the most widely cultivated species among 53 *Gossypium* species

^a^According to IUCN [77]: CR, critically endangered; EN, endangered; VU, vulnerable

^b^Mutation rate (%)

^c^Methylation rate (%)

Similar to species selection, it may be essential to identify suitable donor population(s) and then individuals. Assuming that NGV can be used, at least partially, as a proxy for AGV [89], but see [90], prior information on target species’ genetic diversity and structure could be used to select genetically distinct but diverse populations [11]. Next, the most genetically diverse individuals within the target (source) population could be selected [91]. If researchers are interested in the adaptive potential of traits of a target species, they should first select functional SNPs and genome-wide coding region SNPs. Then, to select source populations and individuals, researchers could measure the AGV of the target species [92]. However, researchers should be aware that if the GE individuals to be translocated or reintroduced are too genetically similar to the recipient population, inbreeding will not be mitigated and there will be no increase in fitness [93, 94]. On the other hand, outbreeding depression is likely if the GE individuals are too genetically different from the donor population [95, 96]. In addition, individuals with a high genetic load are not suitable translocation sources [97], so they may not be appropriate source individuals for GE (or genetic rescue).

## The basis for genome editing: genome resources and hurdles to the implementation of CRISPR tools

As GE involves highly precise changes of genome sequences, high-quality genomes are essential prerequisites. Increasing numbers of plant species have been sequenced since the first genome assembly of the model plant *Arabidopsis thaliana* [98]. According to a recent study, genome assemblies have been constructed for nearly 800 terrestrial plant species [99]. However, very few genome assemblies are available for threatened plants. The first genomic assembly for a threatened plant (the epiphytic orchid *Dendrobium officinale*) was published in 2015 [100] (Additional file 1: Table S1), 15 years after the *A. thaliana* assembly. We found that the list of genome assemblies for terrestrial plant species constructed over the last 20 years includes assemblies for just 10 vulnerable, four endangered, and 11 critically endangered plant species (Fig. 1A; Additional file 1: Table S1), accounting for just 0.11, 0.04, and 0.22% of the total numbers of the species included in the IUCN Red List [77], respectively (Fig. 1B). Interestingly, the distribution of these sequenced threatened species largely overlaps with 25 biodiversity hotspots around the world (Fig. 1A) [101].

The precision of GE depends on the quality of genome assemblies. Recent genome sequence development by long-read sequencing platforms, such as those provided by Pacific Biosciences and Oxford Nanopore Technologies, together with improvements in assembly methodology, can generate gap-free reference genomes [102]. This is critical for conservation genomics because gap-free genomes will greatly facilitate the precise manipulation of target genes by editing genomes of threatened plants. Long-read sequencing technology has become the method of choice for generating genome assemblies in many threatened plant species, accounting for approximately half of the published genome assemblies of threatened plant species (Fig. 1C; Additional file 1: Table S1). In addition, a steady decline in sequencing costs is enabling the resequencing of individuals of threatened plants and can be used (for example) to identify large numbers of SNPs, even with limited numbers of individuals. More importantly, it can identify candidate genes and sites that are potentially responsible for reductions in the fitness of threatened plants [103].

Besides the reference genome resources and gene annotation information, there are still bottlenecks for genome editing [104]. This is about whether it is possible to create a transformant of a specific threatened species. It is theoretically possible but key considerations include finding optimal experimental conditions for transformation, securing genome information, addressing off-target effects, and managing regeneration rates especially slow-growing species like many woody plants [105]. To gain insights into mitigating concerns about these challenges, we collate data on which threatened species the current GE technology can be implemented in, and which it cannot (Additional file 1: Table S1). Encouragingly, 23 of the 64 whole genome sequenced threatened species have tried some effort of GE, either regeneration, transformation, or editing target genes (Additional file 1: Table S1). Seven of the whole genome sequenced threatened species have successfully implemented GE by *Agrobacterium* or biolistic-mediated delivery of editing tools with varied efficiency (Table 1). Hence, we believe that GE techniques have opened new avenues for plant conservation and can facilitate plant adaptation and conservation.

## Mitigation of negative effects of new pathogens and pests or climate change by genome editing

As is well known, GE allows new approaches for breeding disease resistance in a variety of crops [106, 107]. Here, we focus on disease or pathogen resistance in wild plants. A famous application of biotechnology in plant conservation is the attempt to restore the American chestnut *Castanea dentata*, which is severely threatened by the chestnut blight fungus *Cryphonectria parasitica*, by introducing a wheat antifungal gene into its genome. This approach has significantly enhanced its resistance [108]. However, despite its great promise, this transgenic American chestnut is still under evaluation by regulatory agencies before its public release [109]. Newly developed GE technology, such as the delivery of editing tools via mRNA or ribonucleoproteins, has the potential to tackle stumbling blocks by generating transgene-free edits [47, 72, 73, 105, 110]. Producing GE plants with this approach could be a solution to concerns associated with the use of genetically modified organisms (GMOs) and could help address important issues such as food security, sustainability, and conservation (Table 1).

In addition, modern climate change is affecting the distribution of plant species globally and increasing local extinction rates [111, 112]. Although climate change may not have driven any species extinct yet, predictions of high extinction rates in the future all involve it, mainly through increases in temperature and rising CO_2_ levels and both the intensity and frequency of drought [113, 114]. To the best of our knowledge, however, there are no reported uses of GE to reduce threatened plants’ sensitivity to climate change, but some successful case studies in endangered animal species might provide a framework for it [115–117]. Thus, GE appears to have a high potential for future application in the conservation and restoration of threatened plants [118].

Small populations are most susceptible to random genetic drift, which raises their risk for extinction through increasing inbreeding, decreasing within-population genetic variation, and increasing among-population genetic differentiation [17, 119]. Increasing probabilities of inbreeding in small populations also raise risks for inbreeding depression, i.e., recessive deleterious genetic variants becoming homozygous and associated potential reductions in fitness [120]. Small populations are highly vulnerable to the increasing anthropogenic spread of pests and diseases around the world, and new GE technology could be applied to mitigate the negative effects, as described in more detail in the next two sections.

## Identifying deleterious mutations for conservation using genome editing

Genetic problems are common in ex situ collections, which raises major complications for restoring species that are extinct in the wild. Therefore, to conserve threatened plant species, it is essential to identify and change deleterious variants. How are deleterious mutations discovered in threatened and endangered plants? Numerous tools are available for identifying deleterious variants, such as PolyPhen-2 (PP2) [121], LRT [122], PROVEAN [123], GERP +  + [124], and SIFT [125] software packages. The purpose, input data type, and performance of these tools have been well documented and compared in two studies [126, 127]. PROVEAN- and SIFT-based analyses have also shown that endangered island endemic species have significantly more deleterious amino acid variants than non-endangered species at heterozygous sites, explaining why they have lower fitness owing to “vulnerable genomes” [128]. In addition, a whole genome resequencing approach with SIFT was recently applied in an investigation of factors responsible for extremely small populations of the newly described maple species *Acer yangbiense*, endemic to Yunnan, China [129]. Numerous deleterious mutations were identified and the study showed that populations with a high frequency of runs of homozygosity had more homozygous deleterious variants.

**Fig. 2:**
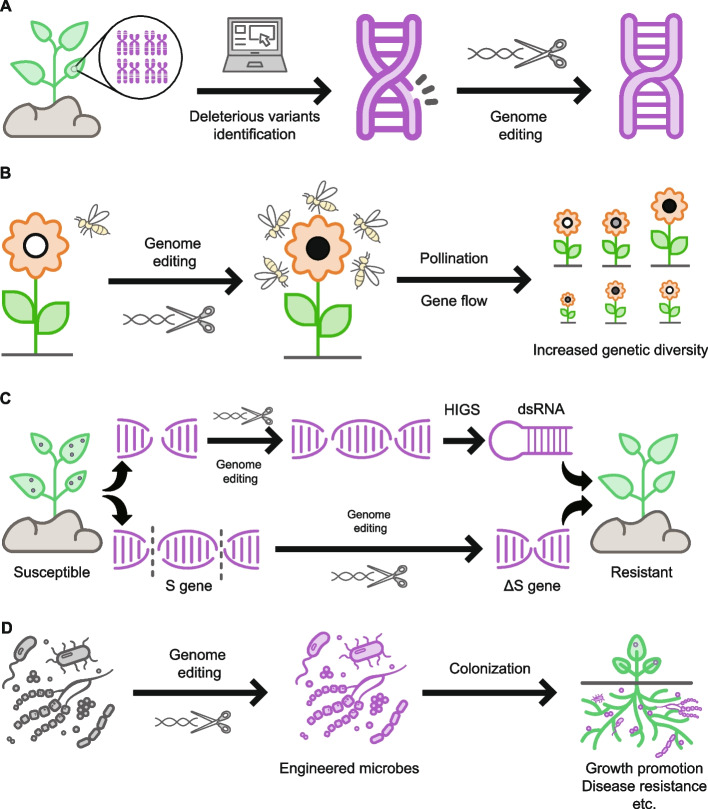
Potential applications of genome editing (GE) in plant conservation. **A** Deleterious mutations can be identified by several software packages at the population level, then modified by GE tools, **B** GE can be used to modify flower pigmentation to increase pollinator visitation, which may lead to increases in gene flow and thus genetic diversity, **C** genomes of susceptible plants can be engineered to enhance their disease resistance either by host induced gene silencing (*HIGS*) or modifying susceptibility (*S*) genes, and **D** GE can be used to engineer plant-associated microbes or microbiomes. The engineered microbes or microbiomes can then colonize either above- or below-ground parts of plants, which may promote resistance, tolerance, and/or growth

Identification and characterization of the sites of such deleterious variants could be highly valuable for assisting the adaptation of threatened species by selecting appropriate GE tools for their precise modification (for details see the “Genome editing tools for plant conservation” section). The edited individuals by correcting deleterious mutations with an adaptive state could potentially spread the adaptive alleles within and between populations by gene flow. The adaptive effects of the resulting GE could then be experimentally evaluated and applied, if successful, in conservation management and restoration (Fig. 2A).

## Maintaining genetic variation by attracting pollinators through genome editing

Gene movement between plant populations is important for maintaining genetic variation and mitigating the negative effects of random genetic drift and inbreeding, which are particularly detrimental for threatened species with fragmented populations [130]. More than 67% of the pollination of flowering plants is mediated by insects, birds, and mammals [131]. Therefore, attracting pollinators is essential for sufficient gene flow among populations of many flowering plants and mitigating the negative effects of random genetic drift and inbreeding depression, especially for threatened species with limited distributions and small populations.

Flowering plants have evolved diverse pollinator-attracting strategies that involve floral pigmentation [132], and increasing evidence has recently revealed their molecular mechanisms. For example, the *HaMYB111* gene in sunflowers is responsible for diverse “bullseye” patterns, with a UV-reflecting ring surrounded by a UV-absorbing ring [133]. A homolog of *HaMYB111* in *Arabidopsis* belongs to a small transcription factor family called PRODUCTION OF FLAVONOL GLYCOSIDES (PFG1/MYB12, PFG2/MYB11, and PFG3/MYB111) that controls the expression of genes involved in the production of flavonol glycosides [134]. These glycosides are the pigments required for floral UV patterns in at least several plant species (e.g., *Brassica rapa* and *Petunia* spp.) [133]. Control of flavonol glycosides by PFG members has also been discovered in other plant species [135, 136]; the PFG family is probably functionally conserved across eudicots [133, 137] and has great potential for modulation of pollinator services.

Accordingly, we propose two strategies to increase the population sizes and mitigate the negative effects of inbreeding depression and random genetic drift on threatened plant species by changing the expression or translation patterns of *PFG* genes and thus pollinator visitation patterns (Fig. 2B). One is to create numerous novel *cis*-regulatory alleles to tailor floral pigmentations by targeting *PFG* promoter regions through GE. This could rapidly and efficiently create a continuum of quantitative trait variation by mutagenizing *cis*-regulatory regions of several developmental genes through multiplexed CRISPR-Cas9 editing, as reported in experiments with tomatoes [138]. An alternative strategy is to manipulate amounts of PFG proteins by editing upstream open reading frames (uORFs), which are short protein-coding elements in the 5′ leader region of downstream primary ORFs (pORFs) [139]. This strategy is based on the discovery that uORF-mediated translational regulation is a general mechanism for controlling protein production [140, 141]. Targeting uORFs by GE can also generate mutants with varying degrees of mRNA translation of the relevant pORFs [142, 143].

## Manipulating microbes indirectly or directly to rescue endangered plant species

### Indirect manipulation

Most plant conservation practices have focused on threatened plants themselves and largely neglected their intimate partners, host-associated microbes [144]. There may be two reasons for this. One is that for a long time, we did not know the key microbes that affect threatened plants’ fitness. The other is that we rarely understand the mechanisms whereby microbes promote or inhibit their growth, development, and reproduction. However, host-associated microbes in the rhizosphere, phyllosphere, and endosphere profoundly influence host plants’ viability (and thus fitness) by regulating their immunity and development [145]. For example, myrtle rust, a disease caused by the fungus *Austropuccinia psidii*, is very invasive, affecting many plants of the large Myrtaceae family [146], *Eugenia gacognei* in New Caledonia [147], and *Rhodomyrtus psidioides* and *R. rubescens* in Australia [148].

The ability of *A. psidii* to attack a very wide range of species, including some with very large populations, suggests that it would not be an easy target, but host susceptibility (*S*) genes have proven value as GE targets to protect crop plants from various pathogens [149]. Thus, targeting the *S* genes required for *A. psidii* invasion is a promising approach for rescuing endangered trees of the Myrtaceae family (Fig. 2C). A possible method is to target homologous *S* genes that are known to confer susceptibility to myrtle rust in well-studied model species. Alternatively, host-induced gene silencing (HIGS) technology [150] could be used to control pathogens by targeting various genes in pathogen genomes. Currently, HIGS relies on random transgenic integration of a double-stranded RNA-producing cassette into a chromosome, so the engineered plants are regarded as GMOs, which may hinder the application of the approach in conservation practices. We propose a strategy involving both HIGS and GE to replace endogenous siRNA or miRNA loci to generate siRNAs targeting pathogens of host plants through prime editing (PE) technology (Fig. 2C; for details see the “Genome editing tools for plant conservation” section). This would generate new lines of threatened plant species without transgenes and thus could be rapidly applied with more potential and less regulation.

### Direct manipulation

In addition to editing plant genomes to indirectly target microbes, GE technology can be harnessed to edit microbes *directly* for plant conservation (Fig. 2D). We focus here on orchids because they account for about a tenth of flowering plants (and thus are one of the largest families), but a large percentage are regarded as threatened or endangered, putatively at least partly due to their complex symbiotic associations with mycorrhizal fungi [151]. All orchid species rely on mycorrhizal fungi for germination, some requiring a specific strain of fungus while others can exploit a broader range of fungi. They also need mycorrhizal fungi in other stages following germination, including flowering. However, the mycorrhization level of different mycorrhizal fungi with orchid seeds or seedlings varies, which may greatly hinder restoration [152]. Interestingly, orchid endophytes forming mycorrhizas are easy to culture [153], so they are good starting materials for engineering by GE to improve their mycorrhization efficiency. An inspiring example is provided by the gene encoding the effector protein SP7 of the arbuscular mycorrhizal fungus *Glomus intraradices* which can efficiently promote mycorrhization [154]. Surprisingly, it has been shown that expression of this gene in the necrotrophic fungus *Magnaporthe oryzae* can attenuate root decay symptoms, suggesting that effectors from mycorrhizal fungi with high mycorrhization levels could be used in those with low levels [154]. This could be accomplished by two GE strategies: using effectors with higher mycorrhization potential to replace endogenous effectors or mimicking their structure using a deep learning-based de novo protein design approach [155].

There is accumulating evidence that plant-associated microbiomes play a pivotal role in plant adaptation [156], so engineering them might rescue endangered or threatened species. Recently, a new approach, dubbed in situ genome engineering, was proposed for manipulating microbial communities in their native contexts, to avoid the difficulties (including low frequencies of culturable taxa) of recapitulating the complex communities found in the field or lab [157, 158]. At least three types of approaches (chemical, cellular, and phage-mediated) can be used to change microbial communities in situ. GE technology has been applied in a phage-mediated approach involving the selective elimination of target strains in a sequence-specific manner via the delivery of a CRISPR-Cas9 system with a bacteriophage [159] and DNA-editing All-in-one RNA-guided CRISPR-Cas Transposase (DART)-mediated targeted GE of microbes using natural transformation [160]. These approaches and derivative versions have high potential utility for manipulating complex microbial communities in situ for conservation purposes (Fig. 2D).

## Challenges facing plant conservation using genome editing

**Fig. 3:**
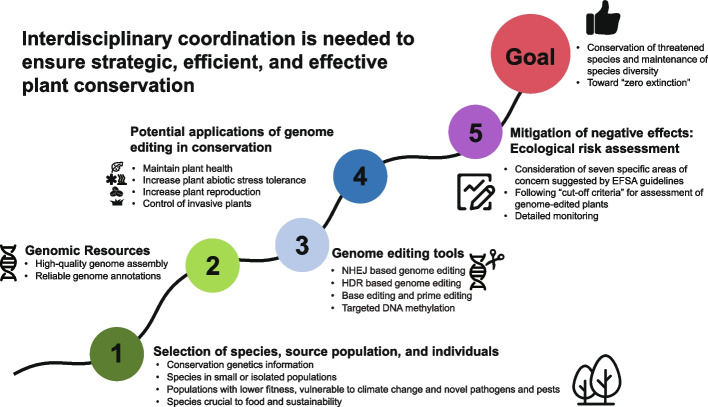
A road map from the feasibility of introducing genome editing (GE) technology to threatened plant species to the creation of successful GE variants

GE technology can provide promising solutions and strategies for plant conservation or restoration (Fig. 3), but there are at least four major challenges. First, as mentioned earlier, obtaining efficient GE transformants is the biggest challenge. The potential of GE is highly dependent on the regeneration efficiency of GE plants, but their genetic transformability is often genotype-dependent. Several developmental regulators (such as WUSCHEL, BABY BOOM, and GROWTH-REGULATING FACTOR) promote the regeneration of some crop plants’ shoots [161], but it is not known if they promote other species’ regeneration in a genotype-independent manner.

Second, most plant GE techniques result in the generation of transgenic plants, which are recognized as GMOs. Thus, their release into natural ecosystems is highly regulated in some countries (Fig. 1A). Although DNA-free strategies can be used to generate mutant plants without leaving GE reagents in the plant genomes [47, 110], their applications are strongly limited by difficulties in protoplast regeneration and marker-free selection of GE plants. Fortunately, a highly promising method that exploits mobile RNA elements for transgene-free GE in wild-type *A. thaliana* and *Brassica rapa* has been recently published [162]. In this method, transgenic rootstocks are initially generated, in which both Cas9 and guide RNA transcripts are fused to tRNA-like sequence motifs. Then, Cas9 and guide RNA move to wild-type shoots grafted onto the transgenic rootstocks and produce transgene-free offspring in a single generation [162]. This method bypasses transgene elimination and tissue culture recovery, which allows its use in a wide range of plants. Currently, the efficiency of inherited homozygotic deletion edits is ca. 0.1%, which may hinder its further application in other non-model plants but this should be improved in the future.

Third, the mating system (self-compatibility, self-incompatibility, autonomous selfing, mixed mating, predominantly outcrossing, apomictic, or exclusively clonally propagation) strongly affects the outcome of plant GE, and it has been mostly applied to inbreeding plants, as homozygous mutants can be obtained by selfing of heterozygous mutants. However, it is extremely difficult to obtain homozygous mutants of outbreeding plants by crossing heterozygous mutants. Therefore, to obtain homozygous mutants of these plants we must increase the GE efficiency to increase the chances of getting homozygous mutants, which are often generated by very small percentages of editing events in the first generation.

Finally, we agree that GE might not provide an instantaneous solution or “panacea/cure-all” to threatened plants but may be part of a long-term conservation strategy [163]. The question of whether genetically edited plants disrupt biodiversity and precipitate ecological crises is also crucial. Like other genetically modified organisms, GE plants for conservation may introduce unexpected risks that harm ecological systems. However, GE plants for conservation are different from those for agricultural uses. For example, GE plants for agricultural uses that are more product-focused can be planted in a restricted area where they have less communication with wild relatives and thus minimize ecological adverse effects. In contrast, once GE plants for conservation are released to their original habitats or other habitats, it may not be possible to control or recall them. Therefore, we must pay more attention to the ecological ramifications of GE plants for conservation. These dangers include risks to non-target organisms (NTOs) and biodiversity, gene flow risks, risks to the evolution of resistance in target species, and weediness risks [164–167]. NTOs impacted by GE plants include beneficial species, non-target herbivores, soil organisms, species of conservation concern, and species contributing to local biodiversity [168]. Gene flow from GE plants might also lead to genetic assimilation in wild relatives, potentially reducing genetic diversity [169]. Although GE plants can introduce new genes to combat pathogens or insects, the evolution of new resistances remains a risk [170, 171]. The persistence or invasiveness of GE plants in natural habitats may cause irreversible effects on biodiversity.

Therefore, the decision framework must include an evaluation of the possibility and degree of potential disruption of the ecosystem and how to manage that disruption [51]. Ecological risk assessments (EcoRA) of GE plants are necessary before their release into nature, which requires data from laboratory tests, field trials, as well as simulation models. The rational approach for EcoRA could be a tiered risk assessment, including hazard/exposure assessment (tier 0), single high dose and dose–response testing (tier 1), refined hazard characterization and exposure assessment (tier 2), further refined risk assessment (tier 3), and additional tiers (tier 4) [172, 173]. This framework should be applied case-by-case because the NTOs’ impact will vary depending on the specific GE plant, the introduced trait, and the environmental conditions where the plant is released. Furthermore, we recommend using an ecologically based method for selecting ecological indicators for EcoRA of GE plants for conservation [174]. Risk assessment for the GE plant should be carefully determined whether the collateral damage (cost) exceeds the benefit. Moreover, recently ecosystem service has been strongly advocated as a key outcome variable in risk assessment in order to connect ecological conservation and human well-being [175].

## Suggested uses of genome-edited plants: reinforcement of declining wild populations

If the risk assessment mentioned above is satisfactory, conservation practitioners may need to implement reinforcement of declining wild populations for focal threatened species. With the help of RONA, GE plants could be used to reinforce declining wild populations of threatened plant taxa *within* target species’ ranges (assisted gene flow) and reintroductions of GE plants at sites where they have disappeared. The establishment of new GE populations *beyond* species’ native ranges (assisted migration) is also a promising approach. Simultaneously, seeds, tissues, DNA, and RNA of GE plants could be deposited in biobanks for further conservation purposes and research [176]. Furthermore, CRISPR-Cas9 gene drives (“selfish genetic elements that are transmitted to progeny at super-Mendelian (> 50%) frequencies” [177]) have promising potential in introducing deleterious edits to populations of invasive plant species for their eradication (Fig. 3). However, using gene drives to push beneficial changes through wild populations of threatened species would be very risky, thus detailed monitoring of populations is essential [178, 179]. It is important to recognize that no approach, including GE, may be suitable for all plant conservation initiatives. Sophisticated simulations should also be applied, if necessary, to select the most appropriate strategies for focal species, populations, and individuals (Fig. 3).

## Concluding remarks and future perspectives

We are aware that not all scientists favor technologically-based solutions, including GE-based approaches. Some believe that genomics should only play a minor role in plant conservation and that primarily we need to reverse the trend of natural destruction with ecologically driven, typically low-tech restoration solutions [180, 181]. However, probably as in the cases of the genetically improved coral *Acropora millepora* [116] and the ongoing mammoth de-extinction project, we expect that the GE approaches described above will soon be successfully applied to threatened plant species. For effective conservation, we recommend the collaboration of molecular biologists engaged in GE research with conservation geneticists, plant ecologists, and conservation practitioners/managers when selecting individuals from natural populations of focal plant taxa for GE. This may help to minimize the negative ecological effects (and maximize the positive effects) of future introductions into the wild. Detailed ecological studies before and after any such introductions may also be highly valuable.

If, in the near future, genetically improved individuals of an endangered species are to be introduced into the wild, it seems appropriate to do so at sites of the source populations because (as stressed in the “Determining species, population(s), and individuals to use for genome editing” section) “local is usually the best” [182, 183]. As our understanding of the underlying biological mechanisms is in its infancy, it is not yet possible to predict with certainty the full range of positive or negative consequences of such GE for organisms at the population to ecosystem level [116]. Therefore, the (re)introduction of GE individuals of endangered species into the wild must be approached with extreme caution. We recommend that they should be initially grown in an isolated botanical garden, experimental station, or uninhabited island, and key traits, including their fitness, should be carefully monitored. Information on how they grow in natural habitats should also be obtained from simulations to minimize probable side effects of GE plants; then they should be carefully reintroduced into ecologically similar habitats (e.g., sites of donor populations) if permitted by relevant authorities.

Lastly, a vital aspect of the recently agreed 2050 Goal A of the Kunming–Montreal Global Biodiversity Framework is the target of “zero extinction.” To achieve this goal, “creating a comprehensive plant inventory, evaluating the conservation status of known species, digitizing herbarium specimens, and preparing tailored recovery plans for threatened species” are crucial [6]. Additionally, removing barriers to conservation caused by a lack of funding and skilled personnel is necessary, and machine learning, citizen science, and new technologies can alleviate this issue [6]. Therefore, to prevent plant extinction on top of this solid infrastructure for conservation, basic and sophisticated ecological research, genetics/genomics research at the species and population level, bioinformatics, and GE approaches that will be developed rapidly and continuously in the future are expected to have a very bright future.

What about the future of GE technology in plant conservation? We believe that a more sophisticated understanding (fundamental and practical) of plants’ resistance and responses to biotic and abiotic stresses is being developed through CRISPR technology, and more robust information will continue to emerge. This seems likely to include functional-genomic information that is highly relevant to both GE and ecological conservation. We further believe that discussing broader issues associated with GE-like technology and its potential application in nature conservation is required to broaden the scope of conservation. These include political and socio-economic issues, raising the need for strongly interdisciplinary GE approaches in conservation.

## Supplementary Information

Additional file 1: Table S1 lists threatened plant species with sequenced genomes.

Additional file 2. Review history.

## References

[1] Barnosky AD, Matzke N, Tomiya S, Wogan GOU, Swartz B, Quental TB, et al. Has the Earth’s sixth mass extinction already arrived? Nature. 2011;471:51–7. 21368823 10.1038/nature09678

[2] Isbell F, Balvanera P, Mori AS, He JS, Bullock JM, Regmi GR, et al. Expert perspectives on global biodiversity loss and its drivers and impacts on people. Front Ecol Environ. 2023;21:94–103.

[3] Humphreys AM, Govaerts R, Ficinski SZ, Nic Lughadha E, Vorontsova MS. Global dataset shows geography and life form predict modern plant extinction and rediscovery. Nat Ecol Evol. 2019;3:1043–7. 31182811 10.1038/s41559-019-0906-2

[4] Ledford H. World’s largest plant survey reveals alarming extinction rate. Nature. 2019;570:148–50. 31186563 10.1038/d41586-019-01810-6

[5] Exposito-Alonso M, Booker TR, Czech L, Gillespie L, Hateley S, Kyriazis C, et al. Genetic diversity loss in the Anthropocene. Science. 2022;377:1431–5. 36137047 10.1126/science.abn5642

[6] Corlett RT. Achieving zero extinction for land plants. Trends Plant Sci. 2023;28:913–23. 37142532 10.1016/j.tplants.2023.03.019

[7] Hamrick JL, Godt MJW. Effects of life history traits on genetic diversity in plant species. Phil Trans R Soc B. 1996;351:1291–8.

[8] Allendorf FW, Hohenlohe PA, Luikart G. Genomics and the future of conservation genetics. Nat Rev Genet. 2010;11:697–709. 20847747 10.1038/nrg2844

[9] Frankham R. Challenges and opportunities of genetic approaches to biological conservation. Conserv Biol. 2010;143:1919–27.

[10] Ottewell KM, Bickerton DC, Byrne M, Lowe AJ. Bridging the gap: a genetic assessment framework for population-level threatened plant conservation prioritization and decision-making. Divers Distrib. 2016;22:174–88.

[11] Chung MY, Merilä J, Li J, Mao K, López-Pujol J, Tsumura Y, et al. Neutral and adaptive genetic diversity in plants: an overview. Front Ecol Evol. 2023;11:1116814.

[12] Heuertz M, Carvalho SB, Galindo J, Rinkevich B, Robakowski P, Aavik T, et al. The application gap: genomics for biodiversity and ecosystem service management. Conserv Biol. 2023;278:09883.

[13] Chung JM, Park KW, Park CS, Lee SH, Chung MG, Chung MY. Contrasting levels of genetic diversity between the historically rare orchid *Cypripedium japonicum* and the historically common orchid *Cypripedium macranthos* in South Korea. Bot J Linn Soc. 2009;160:119–29.

[14] Trapnell DW, Hamrick JL, Negrón-Ortiz V. Genetic diversity within a threatened, endemic North American species, *Euphorbia telephioides* (Euphorbiaceae). Conserv Genet. 2012;13:743–51.

[15] Yang J, Cai L, Liu D, Chen G, Gratzfeld J, Sun W. China’s conservation program on plant species with extremely small populations (PSESP): progress and perspectives. Biol Conserv. 2020;244:108535.

[16] Su J, Yan Y, Song J, Li J, Mao J, Wang N, et al. Recent fragmentation may not alter genetic patterns in endangered long-lived species: evidence from Taxus cuspidata. Front Plant Sci. 2018;9:1571. 30429863 10.3389/fpls.2018.01571 PMC6220038

[17] Ellstrand NC, Elam DR. Population genetic consequences of small population size: implications for plant conservation. Annu Rev Ecol Syst. 1993;24:217–42.

[18] Godt MJW, Walker J, Hamrick JL. Genetic diversity in the endangered lily Harperocallis flava and a close relative. Tofieldia racemosa Conserv Biol. 1997;11:361–6.

[19] Hewitt GM. The genetic legacy of the Quaternary ice ages. Nature. 2000;405:907–13. 10879524 10.1038/35016000

[20] Hewitt GM. Genetic consequences of climatic oscillations in the Quaternary. Phil Trans R Soc B. 2004;359:183–95. 15101575 10.1098/rstb.2003.1388 PMC1693318

[21] Gonzales E, Hamrick JL. Distribution of genetic diversity among disjunct populations of the rare forest understory herb, *Trillium reliquum.* Heredity. 2005;95:306–14. 16094302 10.1038/sj.hdy.6800719

[22] Sork VL, Davis FW, Westfall R, Flint A, Ikegami M, Wang H, et al. Gene movement and genetic association with regional climate gradients in California valley oak (*Quercus lobata* Née) in the face of climate change. Mol Ecol. 2010;19:3806–23. 20723054 10.1111/j.1365-294X.2010.04726.x

[23] Sgrò CM, Lowe AJ, Hoffmann AA. Building evolutionary resilience for conserving biodiversity under climate change. Evol Appl. 2011;4:326–37. 25567976 10.1111/j.1752-4571.2010.00157.x PMC3352557

[24] Hoban SM, Hauffe HC, Pérez-Espona S, Arntzen JW, Bertorelle G, Bryja J, et al. Bringing genetic diversity to the forefront of conservation policy and management. Conserv Genet Resour. 2013;5:593–8.

[25] Hohenlohe PA, Funk WC, Rajora OP. Population genomics for wildlife conservation and management. Mol Ecol. 2021;30:62–82. 33145846 10.1111/mec.15720 PMC7894518

[26] Hoffmann AA, Sgrò CM, Kristensen TN. Revisiting adaptive potential, population size, and conservation. Trends Ecol Evol. 2017;32:506–17. 28476215 10.1016/j.tree.2017.03.012

[27] Petit RJ, Bialozyt R, Brewer S, Cheddadi R, Comps B. From spatial patterns of genetic diversity to postglacial migration processes in forest trees. In: Silvertown J, Antonovics J, editors. Integrating ecology and evolution in a spatial context. Oxford: Blackwell Science; 2001. p. 295–318.

[28] de Villemereuil P, Gaggiotti OE, Mouterde M, Till-Bottraud I. Common garden experiments in the genomic era: new perspectives and opportunities. Heredity. 2016;116:249–54. 26486610 10.1038/hdy.2015.93 PMC4806574

[29] Sork VL. Genomic studies of local adaptation in natural plant populations. J Hered. 2018;109:3–15. 10.1093/jhered/esx091 29045754

[30] Du FK, Wang T, Wang Y, Ueno S, de Lafontaine G. Contrasted patterns of local adaptation to climate change across the range of an evergreen oak, *Quercus aquifolioides.* Evol Appl. 2020;13:2377–91. 33005228 10.1111/eva.13030 PMC7513717

[31] Gugger PF, Fitz-Gibbon ST, Albarrán-Lara A, Wright JW, et al. Landscape genomics of *Quercus lobata* reveals genes involved in local climate adaptation at multiple spatial scales. Mol Ecol. 2021;30:406–23. 33179370 10.1111/mec.15731

[32] Fitzpatrick MC, Keller SR. Ecological genomics meets community-level modelling of biodiversity: mapping the genomic landscape of current and future environmental adaptation. Ecol Lett. 2015;18:1–16. 25270536 10.1111/ele.12376

[33] Rellstab C, Zoller S, Walthert L, Lesur I, Pluess AR, Graf R, et al. Signatures of local adaptation in candidate genes of oaks (*Quercus* spp.) with respect to present and future climatic conditions. Mol Ecol. 2016;25:5907–24. 27759957 10.1111/mec.13889

[34] Bay RA, Harrigan RJ, Underwood VL, Gibbs HL, Smith TB, Ruegg K. Genomic signals of selection predict climate-driven population declines in a migratory bird. Science. 2018;359:83–6. 29302012 10.1126/science.aan4380

[35] Rellstab C, Dauphin B, Exposito-Alonso M. Prospects and limitations of genomic offset in conservation management. Evol Appl. 2021;14:1202–12. 34025760 10.1111/eva.13205 PMC8127717

[36] Feng L, Du FK. Landscape genomics in tree conservation under a changing environment. Front Plant Sci. 2022;95:822217. 10.3389/fpls.2022.822217 PMC8908315 35283901

[37] Borrell JS, Zohren J, Nichols RA, Buggs RJ. Genomic assessment of local adaptation in dwarf birch to inform assisted gene flow. Evol Appl. 2020;13:161–75. 31892950 10.1111/eva.12883 PMC6935589

[38] Rey O, Eizaguirre C, Angers B, Baltazar-Soares M, Sagonas K, Prunier JG, et al. Linking epigenetics and biological conservation: towards a conservation epigenetics perspective. Funct Ecol. 2020;34:414–27.

[39] McGuigan K, Hoffmann AA, Sgrò CM. How is epigenetics predicted to contribute to climate change adaptation? What evidence do we need? Phil Trans R Soc B. 2021;376:20200119. 33866811 10.1098/rstb.2020.0119 PMC8059617

[40] Cong L, Ran FA, Cox D, Lin S, Barretto R, Habib N, et al. Multiplex genome engineering using CRISPR/Cas systems. Science. 2013;339:819–23. 23287718 10.1126/science.1231143 PMC3795411

[41] Mali P, Yang L, Esvelt KM, Aach J, Guell M, DiCarlo JE, et al. RNA-guided human genome engineering via Cas9. Science. 2013;339:823–6. 23287722 10.1126/science.1232033 PMC3712628

[42] Jinek M, East A, Cheng A, Lin S, Ma E, Doudna J. RNA-programmed genome editing in human cells. eLife. 2013;2:e00471. 23386978 10.7554/eLife.00471 PMC3557905

[43] Cho SW, Kim S, Kim JM, Kim JS. Targeted genome engineering in human cells with the Cas9 RNA-guided endonuclease. Nat Biotechnol. 2013;31:230–2. 23360966 10.1038/nbt.2507

[44] Li JF, Norville JE, Aach J, McCormack M, Zhang D, Bush J, et al. Multiplex and homologous recombination–mediated genome editing in *Arabidopsis* and *Nicotiana**benthamiana* using guide RNA and Cas9. Nat Biotechnol. 2013;31:688–91. 23929339 10.1038/nbt.2654 PMC4078740

[45] Nekrasov V, Staskawicz B, Weigel D, Jones JD, Kamoun S. Targeted mutagenesis in the model plant *Nicotiana benthamiana *using Cas9 RNA-guided endonuclease. Nat Biotechnol. 2013;31:691–3. 23929340 10.1038/nbt.2655

[46] Shan Q, Wang Y, Li J, Zhang Y, Chen K, Liang Z, et al. Targeted genome modification of crop plants using a CRISPR-Cas system. Nat Biotechnol. 2013;31:686–8. 23929338 10.1038/nbt.2650

[47] Yin K, Gao C, Qiu JL. Progress and prospects in plant genome editing. Nat Plants. 2017;3:1–6. 10.1038/nplants.2017.107 28758991

[48] Shan S, Soltis PS, Soltis DE, Yang B. Considerations in adapting CRISPR/Cas9 in nongenetic model plant systems. Appl Plant Sci. 2020;8:e11314. 31993256 10.1002/aps3.11314 PMC6976890

[49] Wei W, Gao C. Gene editing: from technologies to applications in research and beyond. Sci China Life Sci. 2022;65:657–9. 35290572 10.1007/s11427-022-2087-5 PMC8922976

[50] Jung C, Till B. Mutagenesis and genome editing in crop improvement: perspectives for the global regulatory landscape. Trends Plant Sci. 2021;26:1258–69. 34465535 10.1016/j.tplants.2021.08.002

[51] Breed MF, Harrison PA, Blyth C, Byrne M, Gaget V, Gellie NJ, et al. The potential of genomics for restoring ecosystems and biodiversity. Nat Rev Genet. 2019;20:615–28. 31300751 10.1038/s41576-019-0152-0

[52] Segelbacher G, Bosse M, Burger P, Galbusera P, Godoy JA, Helsen P, et al. New developments in the field of genomic technologies and their relevance to conservation management. Conserv Genet. 2022;23:217–42.

[53] Buchholzer M, Frommer WB. An increasing number of countries regulate genome editing in crops. New Phytol. 2022;237:12–5. 35739630 10.1111/nph.18333

[54] Huang TK, Puchta H. CRISPR/Cas-mediated gene targeting in plants: finally a turn for the better for homologous recombination. Plant Cell Rep. 2019;38:443–53. 30673818 10.1007/s00299-019-02379-0

[55] Bharat SS, Li S, Li J, Yan L, Xia L. Base editing in plants: current status and challenges. Crop J. 2020;8:384–95.

[56] Molla KA, Sretenovic S, Bansal KC, Qi Y. Precise plant genome editing using base editors and prime editors. Nat Plants. 2021;7:1166–87. 34518669 10.1038/s41477-021-00991-1

[57] Kleinstiver BP, Prew MS, Tsai SQ, Topkar VV, Nguyen NT, Zheng Z, et al. Engineered CRISPR-Cas9 nucleases with altered PAM specificities. Nature. 2015;523:481–5. 26098369 10.1038/nature14592 PMC4540238

[58] Walton RT, Christie KA, Whittaker MN, Kleinstiver BP. Unconstrained genome targeting with near-PAMless engineered CRISPR-Cas9 variants. Science. 2020;368:290–6. 32217751 10.1126/science.aba8853 PMC7297043

[59] Ran FA, Cong L, Yan WX, Scott DA, Gootenberg JS, Kriz AJ, et al. In vivo genome editing using *Staphylococcus aureus *Cas9. Nature. 2015;520:186–91. 25830891 10.1038/nature14299 PMC4393360

[60] Hirano H, Gootenberg JS, Horii T, Abudayyeh OO, Kimura M, Hsu PD, et al. Structure and engineering of *Francisella novicida *Cas9. Cell. 2016;164:950–61. 26875867 10.1016/j.cell.2016.01.039 PMC4899972

[61] Ming M, Ren Q, Pan C, He Y, Zhang Y, Liu S, et al. CRISPR–Cas12b enables efficient plant genome engineering. Nat Plants. 2020;6:202–8. 32170285 10.1038/s41477-020-0614-6

[62] Zetsche B, Gootenberg JS, Abudayyeh OO, Slaymaker IM, Makarova KS, Essletzbichler P, et al. Cpf1 is a single RNA-guided endonuclease of a class 2 CRISPR-Cas system. Cell. 2015;163:759–71. 26422227 10.1016/j.cell.2015.09.038 PMC4638220

[63] Liu JJ, Orlova N, Oakes BL, Ma E, Spinner HB, Baney KL, et al. CasX enzymes comprise a distinct family of RNA-guided genome editors. Nature. 2019;566:218–23. 30718774 10.1038/s41586-019-0908-x PMC6662743

[64] Dolan AE, Hou Z, Xiao Y, Gramelspacher MJ, Heo J, Howden SE, et al. Introducing a spectrum of long-range genomic deletions in human embryonic stem cells using type I CRISPR-Cas. Mol Cell. 2019;74:936–50. 30975459 10.1016/j.molcel.2019.03.014 PMC6555677

[65] Fauser F, Schiml S, Puchta H. Both CRISPR/Cas-based nucleases and nickases can be used efficiently for genome engineering in *Arabidopsis**thaliana*. Plant J. 2014;79:348–59. 24836556 10.1111/tpj.12554

[66] Baltes NJ, Gil-Humanes J, Cermak T, Atkins PA, Voytas DF. DNA replicons for plant genome engineering. Plant Cell. 2014;26:151–63. 24443519 10.1105/tpc.113.119792 PMC3963565

[67] Čermák T, Baltes NJ, Čegan R, Zhang Y, Voytas DF. High-frequency, precise modification of the tomato genome. Genome Biol. 2015;16:1–15. 26541286 10.1186/s13059-015-0796-9 PMC4635538

[68] Wang M, Lu Y, Botella JR, Mao Y, Hua K, Zhu JK. Gene targeting by homology-directed repair in rice using a geminivirus-based CRISPR/Cas9 system. Mol Plant. 2017;10:1007–10. 28315751 10.1016/j.molp.2017.03.002

[69] Sun Y, Zhang X, Wu C, He Y, Ma Y, Hou H, et al. Engineering herbicide-resistant rice plants through CRISPR/Cas9-mediated homologous recombination of acetolactate synthase. Mol Plant. 2016;9:628–31. 26768120 10.1016/j.molp.2016.01.001

[70] Li S, Li J, Zhang J, Du W, Fu J, Sutar S, et al. Synthesis-dependent repair of Cpf1-induced double strand DNA breaks enables targeted gene replacement in rice. J Exp Bot. 2018;69:4715–21. 29955893 10.1093/jxb/ery245 PMC6137971

[71] Komor AC, Kim YB, Packer MS, Zuris JA, Liu DR. Programmable editing of a target base in genomic DNA without double-stranded DNA cleavage. Nature. 2016;533:420–4. 27096365 10.1038/nature17946 PMC4873371

[72] Rees HA, Liu DR. Base editing: precision chemistry on the genome and transcriptome of living cells. Nat Rev Genet. 2018;19:770–88. 30323312 10.1038/s41576-018-0059-1 PMC6535181

[73] Kurt IC, Zhou R, Iyer S, Garcia SP, Miller BR, Langner LM, et al. CRISPR C-to-G base editors for inducing targeted DNA transversions in human cells. Nat Biotechnol. 2021;39:41–6. 32690971 10.1038/s41587-020-0609-x PMC7854778

[74] Zhao D, Li J, Li S, Xin X, Hu M, Price MA, et al. Glycosylase base editors enable C-to-A and C-to-G base changes. Nat Biotechnol. 2021;39:35–40. 32690970 10.1038/s41587-020-0592-2

[75] Gaudelli NM, Komor AC, Rees HA, Packer MS, Badran AH, Bryson DI, et al. Programmable base editing of A• T to G• C in genomic DNA without DNA cleavage. Nature. 2017;551:464–71. 29160308 10.1038/nature24644 PMC5726555

[76] Anzalone AV, Randolph PB, Davis JR, Sousa AA, Koblan LW, Levy JM, et al. Search-and-replace genome editing without double-strand breaks or donor DNA. Nature. 2019;576:149–57. 31634902 10.1038/s41586-019-1711-4 PMC6907074

[77] IUCN (International Union for the Conservation of Nature). IUCN red list of threatened species. Version 2022–2*.* 2022. Available from: https://www.iucnredlist.org

[78] Kui L, Chen H, Zhang W, He S, Xiong Z, Zhang Y, et al. Building a genetic manipulation tool box for orchid biology: identification of constitutive promoters and application of CRISPR/Cas9 in the orchid, *Dendrobium officinale*. Front Plant Sci. 2017;7:2036. 28127299 10.3389/fpls.2016.02036 PMC5226938

[79] Lee JH, Pijut PM. Isolation and characterization of a floral homeotic gene in *Fraxinus**nigra* causing earlier flowering and homeotic alterations in transgenic Arabidopsis. Plant Gene. 2017;10:17–25.

[80] Nagle MF, Nahata SS, Zahl B, Niño de Rivera A, Tacker XV, Elorriaga E, et al. Knockout of floral and meiosis genes using CRISPR/Cas9 produces male-sterility in *Eucalyptus *without impacts on vegetative growth. Plant Direct. 2023;7:e507. 37456612 10.1002/pld3.507 PMC10345981

[81] Newton CB, Young EM, Roberts SC. Targeted control of supporting pathways in paclitaxel biosynthesis with CRISPR-guided methylation. Front Bioeng Biotechnol. 2023;11:1272811. 37915547 10.3389/fbioe.2023.1272811 PMC10616794

[82] Brym M, Brewer S, Wu X, Chambers AH. CRISPR/Cas9-mediated editing of the phytoene desaturase gene in *Vanilla**planifolia* enabling targeted domestication. J Hortic Sci Biotech. 2023;1–10. 10.1080/14620316.2023.2297233.

[83] Cameron KM. Plant ecology: vanilla lures both insects and mammals to disperse its seeds and fruits. Curr Biol. 2023;33:R63–4. 36693309 10.1016/j.cub.2022.12.012

[84] Procko C, Wong WM, Patel J, Mousavi SAR, Dabi T, Duque M, et al. Mutational analysis of mechanosensitive ion channels in the carnivorous Venus flytrap plant. Curr Biol. 2023;33:3257–64. 37437572 10.1016/j.cub.2023.06.048 PMC10528943

[85] Wang P, Zhang J, Sun L, Ma Y, Xu J, Liang S, et al. High efficient multisites genome editing in allotetraploid cotton (*Gossypium**hirsutum*) using CRISPR/Cas9 system. Plant Biotechnol J. 2018;16:137–50. 28499063 10.1111/pbi.12755 PMC5785356

[86] Chen X, Lu X, Shu N, Wang S, Wang J, Wang D, et al. Targeted mutagenesis in cotton (*Gossypium hirsutum *L.) using the CRISPR/Cas9 system. Sci Rep. 2017;7:44304. 28287154 10.1038/srep44304 PMC5347080

[87] Khan Z, Khan SH, Ahmed A, Iqbal MU, Mubarik MS, Ghouri MZ, et al. Genome editing in cotton: challenges and opportunities. J Cotton Res. 2023;6:1–21.

[88] Shim J, Mangat PK, Angeles-Shim RB. Natural variation in wild *Gossypium* species as a tool to broaden the genetic base of cultivated cotton. J Plant Sci Curr Res. 2018;2:005.

[89] DeWoody JA, Harder AM, Mathur S, Willoughby JR. The long-standing significance of genetic diversity in conservation. Mol Ecol. 2021;30:4147–54. 34191374 10.1111/mec.16051

[90] Teixeira JC, Huber CD. The inflated significance of neutral genetic diversity in conservation genetics. Proc Natl Acad Sci USA. 2021;118:e2015096118. 10.1073/pnas.2015096118. 33608481 10.1073/pnas.2015096118 PMC7958437

[91] Ralls K, Sunnucks P, Lacy RC, Frankham R. Genetic rescue: a critique of the evidence supports maximizing genetic diversity rather than minimizing the introduction of putatively harmful genetic variation. Biol Conserv. 2020;251:108784.

[92] He X, Johansson ML, Heath DD. Role of genomics and transcriptomics in selection of reintroduction source populations. Conserv Biol. 2016;30:1010–8. 26756292 10.1111/cobi.12674

[93] Hedrick PW, Garcia-Dorado A. Understanding inbreeding depression, purging, and genetic rescue. Trends Ecol Evol. 2016;31:940–52. 27743611 10.1016/j.tree.2016.09.005

[94] Flanagan SP, Forester BR, Latch EK, Aitken SN, Hoban S. Guidelines for planning genomic assessment and monitoring of locally adaptive variation to inform species conservation. Evol Appl. 2018;11:1035–52. 30026796 10.1111/eva.12569 PMC6050180

[95] Fenster CB, Dudash MR. Genetic considerations for plant population restoration and conservation. In: Bowles J, Whelan CJ, editors. Restoration of endangered species: conceptual issues, planning and implementation. Cambridge: Cambridge University Press; 1994. p. 34–62.

[96] Whitlock R, Stewart GB, Goodman SJ, Piertney SB, Butlin RK, Pullin AS, et al. A systematic review of phenotypic responses to between-population outbreeding. Environ Evid. 2013;2:1–21.

[97] Kyriazis CC, Wayne RK, Lohmueller KE. Strongly deleterious mutations are a primary determinant of extinction risk due to inbreeding depression. Evol Lett. 2021;5:33–47. 33552534 10.1002/evl3.209 PMC7857301

[98] Kaul S, Koo HL, Jenkins J, Rizzo M, Rooney T, Tallon LJ, et al. Analysis of the genome sequence of the flowering plant *Arabidopsis thaliana*. Nature. 2000;408:796–815. 11130711 10.1038/35048692

[99] Marks RA, Hotaling S, Frandsen PB, VanBuren R. Representation and participation across 20 years of plant genome sequencing. Nat Plants. 2021;7:1571–8. 34845350 10.1038/s41477-021-01031-8 PMC8677620

[100] Yan L, Wang X, Liu H, Tian Y, Lian J, Yang R, et al. The genome of *Dendrobium**officinale *illuminates the biology of the important traditional Chinese orchid herb. Mol Plant. 2015;8:922–34. 25825286 10.1016/j.molp.2014.12.011

[101] Myers N, Mittermeier RA, Mittermeier CG, Da Fonseca GA, Kent J. Biodiversity hotspots for conservation priorities. Nature. 2000;403:853–8. 10706275 10.1038/35002501

[102] Song JM, Xie WZ, Wang S, Guo YX, Koo DH, Kudrna D, et al. Two gap-free reference genomes and a global view of the centromere architecture in rice. Mol Plant. 2021;14:1757–67. 34171480 10.1016/j.molp.2021.06.018

[103] Tigano A, Friesen VL. Genomics of local adaptation with gene flow. Mol Ecol. 2016;10:2144–64. 10.1111/mec.13606 26946320

[104] Gao C. The future of CRISPR technologies in agriculture. Nat Rev Mol Cell Biol. 2018;19(275–276):64B. 10.1038/nrm.2018.2 29382940

[105] Mao Y, Botella JB, Liu Y, Zhu JK. Gene editing in plants: progress and challenges. Natl Sci Rev. 2019;6:421–37. 34691892 10.1093/nsr/nwz005 PMC8291443

[106] Schenke D, Cai D. Applications of CRISPR/Cas to improve crop disease resistance: beyond inactivation of susceptibility factors. iScience. 2020;23:101478. 32891884 10.1016/j.isci.2020.101478 PMC7479627

[107] Ledford H. CRISPR-edited crops break new ground in Africa. Nature. 2024;626:245–6. 38278939 10.1038/d41586-024-00176-8

[108] Merkle SA, Andrade GM, Nairn CJ, Powell WA, Maynard CA. Restoration of threatened species: a noble cause for transgenic trees. Tree Genet Genomes. 2007;3:111–8.

[109] Aucott M, Parker RA. Medical biotechnology as a paradigm for forest restoration and introduction of the transgenic American chestnut. Conserv Biol. 2021;35:190–6. 32506503 10.1111/cobi.13566

[110] Woo JW, Kim J, Kwon SI, Corvalán C, Cho SW, Kim S-G, et al. DNA-free genome editing in plants with preassembled CRISPR-Cas9 ribonucleoproteins. Nat Biotech. 2015;33:1162–4. 10.1038/nbt.3389 26479191

[111] Parmesan C, Yohe G. A globally coherent fingerprint of climate change impacts across natural systems. Nature. 2003;421:37–42. 12511946 10.1038/nature01286

[112] Hampe A, Petit RJ. Conserving biodiversity under climate change: the rear edge matters. Ecol Lett. 2005;8:461–7. 21352449 10.1111/j.1461-0248.2005.00739.x

[113] Román-Palacios C, Wiens JJ. Recent responses to climate change reveal the drivers of species extinction and survival. Proc Natl Acad Sci USA. 2020;117:4211–7. 32041877 10.1073/pnas.1913007117 PMC7049143

[114] Massel K, Lam Y, Wong ACS, Hickey LT, Borrell AK, Godwin ID. Hotter, drier, CRISPR: the latest edit on climate change. Theor Appl Genet. 2021;134:1691–709. 33420514 10.1007/s00122-020-03764-0

[115] Piaggio AJ, Segelbacher G, Seddon PJ, Alphey L, Bennett EL, Carlson RH, et al. Is it time for synthetic biodiversity conservation? Trends Ecol Evol. 2017;32:97–107. 27871673 10.1016/j.tree.2016.10.016

[116] Cleves PA, Tinoco AI, Bradford J, Perrin D, Bay LK, Pringle JR. Reduced thermal tolerance in a coral carrying CRISPR-induced mutations in the gene for a heat-shock transcription factor. Proc Natl Acad Sci USA. 2020;117:28899–905. 33168726 10.1073/pnas.1920779117 PMC7682433

[117] Adams WM, Redford KH. Editing the wild. Conserv Biol. 2021;35:1701–3. 10.1111/cobi.13741 33821525

[118] Corlett RT. A bigger toolbox: biotechnology in biodiversity conservation. Trends Biotechnol. 2017;35:55–65. 27424151 10.1016/j.tibtech.2016.06.009

[119] Frankham R. Relationship of genetic variation to population size in wildlife. Conserv Biol. 1996;10:1500–8.

[120] Hedrick PW, Kalinowski ST. Inbreeding depression in conservation biology. Annu Rev Ecol Syst. 2000;31:139–62.

[121] Adzhubei IA, Schmidt S, Peshkin L, Ramensky VE, Gerasimova A, Bork P, et al. A method and server for predicting damaging missense mutations. Nat Methods. 2010;7:248–9. 20354512 10.1038/nmeth0410-248 PMC2855889

[122] Chun S, Fay JC. Identification of deleterious mutations within three human genomes. Genome Res. 2009;19:1553–61. 19602639 10.1101/gr.092619.109 PMC2752137

[123] Choi Y, Chan AP. PROVEAN web server: a tool to predict the functional effect of amino acid substitutions and indels. Bioinformatics. 2015;31:2745–7. 25851949 10.1093/bioinformatics/btv195 PMC4528627

[124] Davydov EV, Goode DL, Sirota M, Cooper GM, Sidow A, Batzoglou S. Identifying a high fraction of the human genome to be under selective constraint using GERP++. PLoS Computat Biol. 2010;6:e1001025. 10.1371/journal.pcbi.1001025 PMC2996323 21152010

[125] Ng PC, Henikoff S. SIFT: predicting amino acid changes that affect protein function. Nucl Acids Res. 2003;31:3812–4. 12824425 10.1093/nar/gkg509 PMC168916

[126] Grimm DG, Azencott CA, Aicheler F, Gieraths U, MacArthur DG, Samocha KE, et al. The evaluation of tools used to predict the impact of missense variants is hindered by two types of circularity. Hum Mutat. 2015;36:513–23. 25684150 10.1002/humu.22768 PMC4409520

[127] Kono TJ, Lei L, Shih CH, Hoffman PJ, Morrell PL, Fay JC. Comparative genomics approaches accurately predict deleterious variants in plants. G3 (Bethesda). 2018;8:3321–9. 30139765 10.1534/g3.118.200563 PMC6169392

[128] Hamabata T, Kinoshita G, Kurita K, Cao PL, Ito M, Murata J, et al. Endangered island endemic plants have vulnerable genomes. Commun Biol. 2019;2:1–10. 31263788 10.1038/s42003-019-0490-7 PMC6597543

[129] Ma Y, Liu D, Wariss HM, Zhang R, Tao L, Milne RI, et al. Demographic history and identification of threats revealed by population genomic analysis provide insights into conservation for an endangered maple. Mol Ecol. 2022;31:767–79. 34826164 10.1111/mec.16289

[130] Ksiazek-Mikenas K, Fant JB, Skogen KA. Pollinator-mediated gene flow connects green roof populations across the urban matrix: a paternity analysis of the self-compatible forb Penstemon hirsutus. Front Ecol Evol. 2019;7:299.

[131] Kearns CA, Inouye DW. Pollinators, flowering plants, and conservation biology. Bioscience. 1997;47:297–307.

[132] Givnish TJ. Ecology of plant speciation. Taxon. 2010;59:1326–66.

[133] Todesco M, Bercovich N, Kim A, Imerovski I, Owens GL, Ruiz ÓD, et al. Genetic basis and dual adaptive role of floral pigmentation in sunflowers. eLife. 2022;11:e72072. 35040432 10.7554/eLife.72072 PMC8765750

[134] Stracke R, Ishihara H, Huep G, Barsch A, Mehrtens F, Niehaus K, et al. Differential regulation of closely related R2R3-MYB transcription factors controls flavonol accumulation in different parts of the *Arabidopsis thaliana *seedling. Plant J. 2007;50:660–77. 17419845 10.1111/j.1365-313X.2007.03078.x PMC1976380

[135] Sheehan H, Moser M, Klahre U, Esfeld K, Dell’Olivo A, Mandel T, et al. MYB-FL controls gain and loss of floral UV absorbance, a key trait affecting pollinator preference and reproductive isolation. Nat Genet. 2016;48:159–66. 26656847 10.1038/ng.3462

[136] Brock MT, Lucas LK, Anderson NA, Rubin MJ, Cody Markelz RJ, Covington MF, et al. Genetic architecture, biochemical underpinnings and ecological impact of floral UV patterning. Mol Ecol. 2016;25:1122–40. 26800256 10.1111/mec.13542

[137] Schilbert HM, Glover BJ. Analysis of flavonol regulator evolution in the Brassicaceae reveals MYB12, MYB111 and MYB21 duplications and MYB11 and MYB24 gene loss. BMC Genomics. 2022;23:1–17. 35986242 10.1186/s12864-022-08819-8 PMC9392221

[138] Rodríguez-Leal D, Lemmon ZH, Man J, Bartlett ME, Lippman ZB. Engineering quantitative trait variation for crop improvement by genome editing. Cell. 2017;171:470–80. 28919077 10.1016/j.cell.2017.08.030

[139] Calvo SE, Pagliarini DJ, Mootha VK. Upstream open reading frames cause widespread reduction of protein expression and are polymorphic among humans. Proc Natl Acad Sci USA. 2009;106:7507–12. 19372376 10.1073/pnas.0810916106 PMC2669787

[140] Von Arnim AG, Jia Q, Vaughn JN. Regulation of plant translation by upstream open reading frames. Plant Sci. 2014;214:1–12. 24268158 10.1016/j.plantsci.2013.09.006

[141] Xu G, Yuan M, Ai C, Liu L, Zhuang E, Karapetyan S, et al. uORF-mediated translation allows engineered plant disease resistance without fitness costs. Nature. 2017;545:491–4. 28514448 10.1038/nature22372 PMC5532539

[142] Zhang H, Si X, Ji X, Fan R, Liu J, Chen K, et al. Genome editing of upstream open reading frames enables translational control in plants. Nat Biotechnol. 2018;36:894–8. 30080209 10.1038/nbt.4202

[143] Xue C, Qiu F, Wang Y, Li B, Zhao KT, Chen K, et al. Tuning plant phenotypes by precise, graded downregulation of gene expression. Nat Biotechnol. 2023. 10.1038/s41587-023-01707-w. 36894598 10.1038/s41587-023-01707-w

[144] Trevelline BK, Fontaine SS, Hartup BK, Kohl KD. Conservation biology needs a microbial renaissance: a call for the consideration of host-associated microbiota in wildlife management practices. Proc Royal Soc B. 2019;286:20182448. 10.1098/rspb.2018.2448 PMC6364583 30963956

[145] Vorholt JA. Microbial life in the phyllosphere. Nat Rev Microbiol. 2012;10:828–40. 23154261 10.1038/nrmicro2910

[146] Fensham RJ, Carnegie AJ, Laffineur B, Makinson RO, Pegg GS, Wills J. Imminent extinction of Australian Myrtaceae by fungal disease. Trends Ecol Evol. 2020;35:554–7. 32340836 10.1016/j.tree.2020.03.012

[147] Anderegg WR, Kane JM, Anderegg LD. Consequences of widespread tree mortality triggered by drought and temperature stress. Nat Clim Chang. 2013;3:30–6.

[148] Fensham RJ, Radford-Smith J. Unprecedented extinction of tree species by fungal disease. Biol Conserv. 2021;261:109276.

[149] Yin K, Qiu JL. Genome editing for plant disease resistance: applications and perspectives. Phil Trans R Soc B. 2019;374:20180322. 30967029 10.1098/rstb.2018.0322 PMC6367152

[150] Koch A, Wassenegger M. Host-induced gene silencing–mechanisms and applications. New Phytol. 2021;231:54–9. 33774815 10.1111/nph.17364

[151] Fay MF. Orchid conservation: how can we meet the challenges in the twenty-first century? Bot Stud. 2018;59:1–6. 29872972 10.1186/s40529-018-0232-z PMC5988927

[152] Pereira G, Herrera H, Arriagada C, Cid H, García JL, Atala C. Controlled mycorrhization of the endemic Chilean orchid *Chloraea**gavilu* (Orchidaceae). Plant Biosyst. 2021;155:848–55.

[153] Zettler LW, Corey LL. Orchid mycorrhizal fungi: isolation and identification techniques. In: Lee YI, Yeung ET, editors. Orchid propagation: from laboratories to greenhouses—methods and protocols. New York: Humana Press; 2018. p. 27–59. 10.1007/978-1-4939-7771-0_2.

[154] Kloppholz S, Kuhn H, Requena N. A secreted fungal effector of *Glomus**intraradices* promotes symbiotic biotrophy. Curr Biol. 2011;21:1204–9. 21757354 10.1016/j.cub.2011.06.044

[155] Yeh AHW, Norn C, Kipnis Y, Tischer D, Pellock SJ, Evans D, et al. De novo design of luciferases using deep learning. Nature. 2023;614:774–80. 36813896 10.1038/s41586-023-05696-3 PMC9946828

[156] Carthey AJ, Blumstein DT, Gallagher RV, Tetu SG, Gillings MR. Conserving the holobiont. Funct Ecol. 2020;34:764–76.

[157] Sheth RU, Cabral V, Chen SP, Wang HH. Manipulating bacterial communities by in situ microbiome engineering. Trends Genet. 2016;32:189–200. 26916078 10.1016/j.tig.2016.01.005 PMC4828914

[158] Ronda C, Chen SP, Cabral V, Yaung SJ, Wang HH. Metagenomic engineering of the mammalian gut microbiome in situ. Nat Methods. 2019;16:167–70. 30643213 10.1038/s41592-018-0301-y PMC6467691

[159] Bikard D, Euler CW, Jiang W, Nussenzweig PM, Goldberg GW, Duportet X, et al. Exploiting CRISPR-Cas nucleases to produce sequence-specific antimicrobials. Nat Biotechnol. 2014;32:1146–50. 25282355 10.1038/nbt.3043 PMC4317352

[160] Rubin BE, Diamond S, Cress BF, Crits-Christoph A, Lou YC, Borges AL, et al. Species-and site-specific genome editing in complex bacterial communities. Nat Microbiol. 2022;7:34–47. 34873292 10.1038/s41564-021-01014-7 PMC9261505

[161] Nalapalli S, Tunc-Ozdemir M, Sun Y, Elumalai S, Que Q. Morphogenic regulators and their application in improving plant transformation. Methods Mol Biol. 2021;2238:37–61. 33471323 10.1007/978-1-0716-1068-8_3

[162] Yang L, Machin F, Wang S, Saplaoura E, Kragler F. Heritable transgene-free genome editing in plants by grafting of wild-type shoots to transgenic donor rootstocks. Nat Biotechnol. 2023. 10.1038/s41587-022-01585-8. 36593415 10.1038/s41587-022-01585-8 PMC10344777

[163] Brookes G, Smyth SJ. Risk-appropriate regulations for gene-editing technologies. GM Crops Food. 2024;15:1–14. 38215017 10.1080/21645698.2023.2293510 PMC10793663

[164] Snow AA, Palma PM. Commercialization of transgenic plants: potential ecological risks. BioSci. 1997;47:86–96.

[165] Andow DA, Zwahlen C. Assessing environmental risks of transgenic plants. Ecol Lett. 2006;9:196–214. 16958885 10.1111/j.1461-0248.2005.00846.x

[166] Tsatsakis AM, Nawaz MA, Kouretas D, Balias G, Savolainen K, Tutelyan VA, et al. Environmental impacts of genetically modified plants: a review. Environ Res. 2017;156:818–33. 28347490 10.1016/j.envres.2017.03.011

[167] Bauer-Panskus A, Miyazaki J, Kawall K, Then C. Risk assessment of genetically engineered plants that can persist and propagate in the environment. Environ Sci Eur. 2020;32:1–15.

[168] Snow AA, Andow DA, Gepts P, Hallerman EM, Power A, Tiedje JM, et al. Genetically engineered organisms and the environment: current status and recommendations. Ecol Appl. 2005;15:377–404.

[169] Wolfenbarge LL, Phifer PR. The ecological risks and benefits of genetically engineered plants. Science. 2000;290:2088–93. 11118136 10.1126/science.290.5499.2088

[170] Dybdahl MF, Storfer A. Parasite local adaptation: red queen versus suicide king. Trends Ecol Evol. 2003;18:523–30.

[171] Parker IM, Gilbert GS. The evolutionary ecology of novel plant-pathogen interactions. Annu Rev Ecol Evol Syst. 2004;35:675–700.

[172] Poppy G. GM crops: environmental risks and non-target effects. Trends Plant Sci. 2000;5:4–6. 10809593 10.1016/s1360-1385(99)01514-9

[173] Garcia-Alonso M, Jacobs E, Raybould A, Nickson TE, Sowig P, Willekens H, et al. A tiered system for assessing the risk of genetically modified plants to non-target organisms. Environ Biosafety Res. 2006;5:57–65. 17328852 10.1051/ebr:2006018

[174] Andow DA, Lovei GL, Arpaia S, Wilson L, Fontes EM, Hilbeck A, et al. An ecologically-based method for selecting ecological indicators for assessing risks to biological diversity from genetically-engineered plants. J biosaf. 2013;22:141–56.

[175] Munns WR Jr, Rea AW, Suter GW II, Martin L, Blake-Hedges L, Crk T, et al. Ecosystem services as assessment endpoints for ecological risk assessment. Integr Environ Assess Manag. 2016;12:522–8. 26331725 10.1002/ieam.1707

[176] Gao B, Shu Z, Ren S, Gao D. Biobanking: a foundation of life-science research and advancement. Biosaf Health. 2022;4:285–9.

[177] Bier E. Gene drives gaining speed. Nat Rev Genet. 2022;23:5–22. 34363067 10.1038/s41576-021-00386-0 PMC8344398

[178] Barrett LG, Legros M, Kumaran N, Glassop D, Raghu S, Gardiner DM. Gene drives in plants: opportunities and challenges for weed control and engineered resilience. Proc R Soc B. 2019;286:20191515. 31551052 10.1098/rspb.2019.1515 PMC6784734

[179] Sandler R. The ethics of genetic engineering and gene drives in conservation. Conserv Biol. 2019;34:378–85. 31397921 10.1111/cobi.13407

[180] Harrison H, Hauer J, Nielsen J, Aas Ø. Disputing nature in the Anthropocene: technology as friend and foe in the struggle to conserve wild Atlantic salmon (*Salmo salar*). Ecol Soc. 2019;24:13.

[181] Parris-Piper N, Dressler WH, Satizábal P, Fletcher R. Automating violence? The anti-politics of ‘smart technology’ in biodiversity conservation. Biol Conserv. 2023;278:109859.

[182] Aitken SN, Bemmels JB. Time to get moving: assisted gene flow of forest trees. Evol Appl. 2016;9:271–90. 27087852 10.1111/eva.12293 PMC4780373

[183] Chung MY, Son S, Herrando-Moraira S, Tang C, Maki M, Kim Y-D, et al. Incorporating differences between genetic diversity of trees and herbaceous plants in conservation strategies. Conserv Biol. 2020;34:1142–51. 31994789 10.1111/cobi.13467

