# Supplementary material for: Plant conservation in the age of genome editing: opportunities and challenges
Source: Genome Biol. 2024 Oct 24;25:279. doi: 10.1186/s13059-024-03399-0 (PMC11515576; doi:10.1186/s13059-024-03399-0)
Supplement: Supplementary file 2 — Additional file 2. Review history. [file 13059_2024_3399_MOESM2_ESM.docx]

Review History

**First round of review**

**Reviewer 1**

**Comments to author:**

The manuscript "Plant conservation in the age of genome editing: Opportunities and challenges" reports the authors´ efforts to provide an overview of the role of gene editing in plants conversion and the problems that may be encountered. Authors highlights that harnessing gene editing to strengthen the resistance to biotic and abiotic stress, paired with the regulation of pollinator and microbe activity, can significantly aid in the preservation of endangered plants. Furthermore, the article also presents potential challenges in areas such as technology, ecology, and policy that this method might encounter, as well as corresponding solutions. This article can assist readers in gaining a more comprehensive and in-depth understanding of the application and impact of gene editing technology in plant conservation. However, the manuscript has some issues in both content and expression that require careful revision by the author.

Major comments:

1 The availability of experimental conditions for gene editing in endangered species is a crucial issue. This manuscript only mentions that a limited number of threatened species have been sequenced, but it does not provide information on the status of their genome research or the use of genome editing techniques within them.The lack of suitable reference genomes and gene annotation information can make it difficult for researchers to select gene editing target sites. Moreover, there are challenges associated with employing gene editing techniques in these threatened plants, such as delivery and transformation systems, regeneration of genome-edited plants. As we know low transformation and in vitro regeneration abilities, which are exacerbated by their naturally slow growth rates. These bottlenecks pose significant obstacles to the broader application of genome-editing technologies in several specie. In case, gene editing technology cannot be applied to some endangered plants, then discussions about plant conservation become difficult to realize. Therefore, the authors should collate data on which endangered species the current gene-editing technology can be implemented in, and which it cannot. If implementation is not feasible, they should outline the issues faced and assess their complexity.

2 The question of whether genetically edited plants disrupt biodiversity and precipitate ecological crises is also crucial. For example, Cry3Bb Bt-maize might influence two types of beneficial beetles, potentially disrupting the equilibrium of the entire ecosystem. It is imperative for this manuscript to incorporate a detailed examination of this issue. Authors should carefully examine the potential negative impacts of genetically engineered plants on the ecological environment and determine whether the potential collateral damage exceeds the benefits.

3 Many places in the article that mention gene editing lack detailed justification. For example, what is referred to as the newly developed GE technology in line 174, and how is it used to form transgene-free edits and enhance the resistance of endangered plants? The sentence in line 218, "Identification and characterization of the sites of ... for their precise modification", does not elaborate on how to select appropriate gene editing tools, nor does it explain how to use them to eliminate the impact of deleterious mutations in endangered plants. These methods seem theoretically feasible, but the text does not provide information on how to implement them in practice or any potential issues that might arise.

4 The section "Mitigation of negative effects of new pathogens and pests or climate change by genome editing" in the article proposes the use of gene editing to lessen the impact of pests on threatened plants as stated in the title, which holds significant value for plant conservation. However, it does not substantiate this claim later in the text. Moreover, the example of Oryza alta is inappropriate (which I will elaborate on in the next point). Therefore, the examples provided by the author are insufficient to support their point of view. I suggest adding more content and references related to this topic.

5 In lines 187 to 190 of the article, "For example, the successful de novo domestication of Oryza alta ... classified as Near Threatened", the author argues that the de novo domestication of Oryza alta proves that gene editing can protect threatened wild rice species from climate change. However, I think this example does not directly support the author's point. Oryza alta inherently has resistance to abiotic and biotic stresses. In the process of its de novo domestication, gene editing mainly improves important agronomic traits, including seed shattering, awn length, plant height, etc., making it a more suitable cultivated variety. De novo domestication leverages the inherent environmental adaptability of wild rice, rather than reinforcing its adaptability for the purpose of preservation. Therefore, I consider this example to be inappropriate.

Minor comments:

There are also minor grammar and expression errors in the article. For example, in line 514 of the article, "While NHEJ can efficiently generate mutations in target regions (mostly +1/-1 mutations at the -3 position upstream of a PAM site), but not as precisely as HDR" The use of "while" and "but" is redundant, and the sentence could benefit from removing one. Moreover, the "xi" in line 471 of the article should be "ix". Please carefully check the manuscript and revise these points

**Reviewer 2**

**Comments to author:**

The manuscript by Yin et al., entitled "Plant Conservation in the Age of Genome Editing: Opportunities and Challenges," tackles the critical issue of plant conservation for endangered species—a subject of considerable importance. The article is articulate and introduces key concepts effectively. However, I recommend a more structured presentation that clearly defines the conservation challenges, delves deeper into potential solutions, and provides illustrative examples. While recognizing the importance of all plant species, the manuscript would benefit from an emphasis on those crucial to food security and sustainability. The authors should articulate where genomic interventions could have the most significant impact.

Additionally, a historical perspective on extinct and endangered species would enrich the context of the current conservation efforts and the urgency of employing new technologies like genome editing.

Speaking of genome editing, this section of the review is currently insufficient. The authors are advised to consult a genome editing specialist to expand upon the existing tools. A comprehensive list of reagents, along with their specific applications, intended gene targets, and desired outcomes, would greatly enhance the manuscript's utility.

A visual representation, such as a figure or roadmap, outlining the strategic use of gene editing in plant conservation would be highly beneficial. This review should not only inform but also stimulate intellectual curiosity. It should pose challenging questions and suggest innovative solutions, thereby providing readers with a compelling narrative and actionable insights.

**Authors Response**

**Point-by-point responses to the reviewers’ comments:**

Reviewer #1:

The manuscript “Plant conservation in the age of genome editing: Opportunities and challenges” reports the authors’ efforts to provide an overview of the role of gene editing in plants conversion and the problems that may be encountered. Authors highlights that harnessing gene editing to strengthen the resistance to biotic and abiotic stress, paired with the regulation of pollinator and microbe activity, can significantly aid in the preservation of endangered plants. Furthermore, the article also presents potential challenges in areas such as technology, ecology, and policy that this method might encounter, as well as corresponding solutions. This article can assist readers in gaining a more comprehensive and in-depth understanding of the application and impact of gene editing technology in plant conservation. However, the manuscript has some issues in both content and expression that require careful revision by the author.

*Our reply: Thank you for your overall positive comments. We have revised the manuscript to our best to address the major comments.*

Major comments:

1. The availability of experimental conditions for gene editing in endangered species is a crucial issue. This manuscript only mentions that a limited number of threatened species have been sequenced, but it does not provide information on the status of their genome research or the use of genome editing techniques within them. The lack of suitable reference genomes and gene annotation information can make it difficult for researchers to select gene editing target sites. Moreover, there are challenges associated with employing gene editing techniques in these threatened plants, such as delivery and transformation systems, regeneration of genome-edited plants. As we know low transformation and in vitro regeneration abilities, which are exacerbated by their naturally slow growth rates. These bottlenecks pose significant obstacles to the broader application of genome-editing technologies in several species. In case, gene editing technology cannot be applied to some endangered plants, then discussions about plant conservation become difficult to realize. Therefore, the authors should collate data on which endangered species the current gene-editing technology can be implemented in, and which it cannot. If implementation is not feasible, they should outline the issues faced and assess their complexity.

*Our reply: Thank you for your critical but helpful comments on our manuscript. We agree that, besides the reference genome resources and gene annotation information, the way how to employ genome editing (GE) for endangered species is critical. Therefore, we have restudied all the published GE statuses for the threatened plant species with reference genomes. To our surprise, we found that 23 out of 64 empirical studies have tried some efforts to the road map of GE. Seven of the whole genome sequenced threatened species have successfully implemented GE by Agrobacterium or biolistic-mediated delivery of editing tools, and the other 16 species have either established regeneration or genetic transformation methods required for GE implementation in threatened plants. We agree that GE might not be suitable for all endangered species but at least one-third of the sequenced threatened species have tried GE to some extent, it indeed provides a way to dig out the endangered mechanism and guides the protection strategy of threatened species. This information has been added to a new column in Additional Table S1 and in the revised version we have discussed the challenges of GE in endangered species.*

*Furthermore, to reflect your comments comprehensively we have changed the title of “The basis for genome editing: Genome resources” to “The basis for genome editing: Genome resources and hurdles to the implementation of CRISPR tools” and, accordingly, have expanded the section.*

2. The question of whether genetically edited plants disrupt biodiversity and precipitate ecological crises is also crucial. For example, Cry3Bb Bt-maize might influence two types of beneficial beetles, potentially disrupting the equilibrium of the entire ecosystem. It is imperative for this manuscript to incorporate a detailed examination of this issue. Authors should carefully examine the potential negative impacts of genetically engineered plants on the ecological environment and determine whether the potential collateral damage exceeds the benefits.

*Our reply: Yes, we fully agree with your suggestions. Accordingly, in the revised version, we have included the example of the Cry3Bb Bt-maize story (Stephens et al., 2012) as a final case in the section “Challenges facing plant conservation using genome editing”. Furthermore, in the revised version, we have recommended that researchers follow the targets set in the Global Biodiversity Framework (GBF), agreed upon by the end of 2022. As we agree that “applying gene editing technologies will not provide an instantaneous solution, but will be part of a long-term strategy” (Brookes and Smyth, 2024), we have added this information to the section “Challenges facing plant conservation using genome editing” to encourage long-term research and to avoid or mitigate disruption of the balance of the overall ecosystem. In the revised version, we have also briefly introduced the “seven stages of risk assessment” for GE plants proposed by the European Food Safety Authority (EFSA) and newly proposed “cut-off criteria” (EFSA GMO Panel, 2022; Bauer-Panskus et al., 2020). In addition, we have strongly felt to introduce the present situation that forest certification bodies (e.g., the Forest Stewardship Council in Bonn, Germany; the Program for the Endorsement of Forest Certification in Geneva, Switzerland) have consistently excluded GE trees from certification (Strauss et al., 2019). Based on personal communication with Professor Steve Strauss, “Currently, there is very little public or private sector activity worldwide, and extremely few field trials, directed to science and technology in support of commercial uses of GE or genetically engineered (GEN) forest trees. this rejection is because of the absolute restriction on the use of GEN forest trees in commercial products under all of the international and major national forest certification systems.” We have included his opinion in the revised manuscript.*

*References:*

*EFSA GMO Panel (EFSA Panel on Genetically Modified Organisms), Mullins E, Bresson J-L, Dalmay T, Dewhurst IC, Epstein MM, Firbank LG, et al. Statement on criteria for risk assessment of plants produced by targeted mutagenesis, cisgenesis and intragenesis. EFSA Journal. 2022;20:7618, 12 pp.*

*Bauer-Panskus, A., Miyazaki, J., Kawall, K., & Then, C. (2020). Risk assessment of genetically engineered plants that can persist and propagate in the environment. Environmental Sciences Europe, 32(1), 1-15.*

*Brookes G, Smyth SJ. Risk-appropriate regulations for gene-editing technologies. GM Crops Food. 2024;15:1-14.*

*Stephens EJ, Losey JE, Allee LL, DiTommaso A, Bodner C, Breyre A. The impact of Cry3Bb Bt-maize on two guilds of beneficial beetles. Agric Ecosyst Environ. 2012;156:72-81.*

*Strauss SH, Boerjan W, Chiang V, Costanza A, Coleman H, Davis JM, et al. Certification for gene-edited forests. Science. 2019;365:767-8.*

3. Many places in the article that mention gene editing lack detailed justification. For example, what is referred to as the newly developed GE technology in line 174, and how is it used to form transgene-free edits and enhance the resistance of endangered plants? The sentence in line 218, “Identification and characterization of the sites of ... for their precise modification”, does not elaborate on how to select appropriate gene editing tools, nor does it explain how to use them to eliminate the impact of deleterious mutations in endangered plants. These methods seem theoretically feasible, but the text does not provide information on how to implement them in practice or any potential issues that might arise.

*Our reply: For clarity on the “newly developed GE technology”, we have revised this part by adding a new reference (Mao et al., 2019): “Newly developed GE technology, such as the delivery of editing tools via mRNA or ribonucleoproteins (RNP), has the potential to tackle this stumbling blocks by generating transgene-free edits. We strongly feel the need for the revision of the second paragraph (“Identification and characterization of the sites …… GE tools for their precise modification”) in the section “Identifying deleterious mutations for conservation using genome editing”. As we put the description of GE tools in Box 2, NHEJ (non-homologous end joining)-based gene editing, HDR (homology-directed repair)-based gene editing, prime editing and base editing generate small indels, large insertions, short insertions and point mutations, respectively. Therefore, in the revised version, we have changed this sentence to “Identification and characterization of the sites of such deleterious variants could be highly valuable for assisting the adaptation of threatened species by selecting appropriate GE tools for their precise modification (see Box 2 for details)”. The Reviewer #1 recommends our explanation on “how to use them to eliminate the impact of deleterious mutations in endangered plants”. Since the editing of deleterious mutations to an adaptive state could potentially increase the edited individual’s adaptability (and thus, fitness), and this potential adaptability can be sustained or spread out by gene flow within and between populations, thus we can potentially help to conserve the populations and the species. In the revised version, we thus have added the sentence “The edited individuals by correcting deleterious mutations with an adaptive state could potentially spread the adaptive alleles within and between populations by gene flow.” As the “potential issues” are the common questions faced by conservation practice using GE, we have discussed these issues in the section “Challenges facing plant conservation using genome editing”.*

*Reference:*

*Mao Y, Botella JB, Liu Y, Zhu J-K. Gene editing in plants: progress and challenges. Natl Sci Rev.2019;6:421-37.*

4. The section “Mitigation of negative effects of new pathogens and pests or climate change by genome editing” in the article proposes the use of gene editing to lessen the impact of pests on threatened plants as stated in the title, which holds significant value for plant conservation. However, it does not substantiate this claim later in the text. Moreover, the example of Oryza alta is inappropriate (which I will elaborate on in the next point). Therefore, the examples provided by the author are insufficient to support their point of view. I suggest adding more content and references related to this topic.

*Our reply: Thanks for your constructive suggestions. Accordingly, we have added more content and references. Yes, we agree that “the example of Oryza alta is inappropriate” because gene editing indeed improves the agronomic traits of O. alta but does not directly protect the wild rice species. Therefore, we have deleted this part. See also “Our reply” to comment #5 below.*

5. In lines 187 to 190 of the article, “For example, the successful de novo domestication of Oryza alta ... classified as Near Threatened”, the author argues that the de novo domestication of Oryza alta proves that gene editing can protect threatened wild rice species from climate change. However, I think this example does not directly support the author’s point. Oryza alta inherently has resistance to abiotic and biotic stresses. In the process of its de novo domestication, gene editing mainly improves important agronomic traits, including seed shattering, awn length, plant height, etc., making it a more suitable cultivated variety. De novo domestication leverages the inherent environmental adaptability of wild rice, rather than reinforcing its adaptability for the purpose of preservation. Therefore, I consider this example to be inappropriate.

*Our reply: In the revised version, we have replaced the example of Oryza alta with the genome-edited rescued endangered animal case study. Besides the successful case study of corals, we added more conservation applications of gene editing in several animals even some of them in the proposal stage (Piaggio et al., 2017; Adams and Redford, 2021 and for several animals).*

*References:*

*Piaggio AJ, Segelbacher G, Seddon PJ, Alphey L, Bennett EL, Carlson RH, et al. Is it time for synthetic biodiversity conservation? Trends Ecol Evol. 2017;32:97-107.*

*Adams WM, Redford KH. Editing the wild. Conserv Biol. 2021;1-3.*

Minor comments:

There are also minor grammar and expression errors in the article. For example, in line 514 of the article, “While NHEJ can efficiently generate mutations in target regions (mostly +1/-1 mutations at the -3 position upstream of a PAM site), but not as precisely as HDR” The use of “while” and “but” is redundant, and the sentence could benefit from removing one. Moreover, the “xi” in line 471 of the article should be “ix”. Please carefully check the manuscript and revise these points.

*Our reply: We apologize for any minor grammar and expression errors. We did our best to eliminate such errors when revising the manuscript. The English of the manuscript is edited by a professional language editor (www.sees-editing.co.uk) before resubmission.*

Reviewer #2:

The manuscript by Yin et al., entitled “Plant Conservation in the Age of Genome Editing: Opportunities and Challenges,” tackles the critical issue of plant conservation for endangered species—a subject of considerable importance. The article is articulate and introduces key concepts effectively. However, I recommend a more structured presentation that clearly defines the conservation challenges, delves deeper into potential solutions, and provides illustrative examples. While recognizing the importance of all plant species, the manuscript would benefit from an emphasis on those crucial to food security and sustainability. The authors should articulate where genomic interventions could have the most significant impact.

*Our reply: Thank you for your positive comments and insightful suggestions. Using the seven of the whole genome sequenced threatened species successfully implemented genome-editing (GE) as an example, in the revised version we have clearly defined their conservation challenges or implications (see the new Table 1). In recent years, many attempts have been made to establish gene editing systems and demonstrate their efficiency in trait stacking, abiotic stress tolerance improvement, and hybrid breeding, we have described this in detail in the column “Main motivation or purpose of GE” in Table 1. In connection with this issue, in the revised version we have included the first paragraph in the section “Defining species, population(s), and individuals to use for GE” with a discussion and recommendation on the basis for which researchers select plants to be GE.*

*Since GE’s attempts will help to address the important issues of food security and sustainability, we have included this information in the revised illustration (Fig 3). As Reviewer #1 also suggested, in the last paragraph in the section “Challenges facing plant conservation using GE” we have included the scenario of the negative influence caused by Cry3Bb Bt-maize on the two beneficial beetles. As a similar situation could occur in GE plants, we have also suggested potential researchers have promising solutions by following “the seven stages of risk assessment” for GE plants proposed by the European Food Safety Authority (EFSA) and newly proposed “cut-off criteria” (Bauer-Panskus et al., 2020; EFSA GMO Panel, 2022).*

*References:*

*EFSA GMO Panel (EFSA Panel on Genetically Modified Organisms), Mullins E, Bresson J-L, Dalmay T, Dewhurst IC, Epstein MM, Firbank LG, et al. Statement on criteria for risk assessment of plants produced by targeted mutagenesis, cisgenesis and intragenesis. EFSA Journal. 2022;20:7618, 12 pp.*

*Bauer-Panskus A, Miyazaki J, Kawall K, Then C. Risk assessment of genetically engineered plants that can persist and propagate in the environment. Environ Sci Eur. 2020; 32:1-15.*

Additionally, a historical perspective on extinct and endangered species would enrich the context of the current conservation efforts and the urgency of employing new technologies like genome editing.

*Our reply: Thank you for your important comment. In the Introduction, we already included briefly a historical perspective of traditional conservation genetics and recent conservation genomics and explained why GE is needed for the conservation of threatened plants. The historical perspectives and attempts at conservation by synthetic biology of extinct and endangered species have been the object of some review articles (Adams and Redford, 2021; Novak, 2018; Piaggio et al., 2017). For reference, we have added the sentence in the first paragraph of the Introduction in the revised text: “Recent studies have confirmed that the sixth extinction event began approximately 100,000 years ago, coinciding with the migration of modern humans to different parts of the world, and is still ongoing (Barnosky et al., 2011).”*

*References:*

*Adams WM, Redford KH. Editing the wild. Conserv Biol. 2021;1-3.*

*Novak BJ. De-extinction. Genes. 2018;9:548.*

*Piaggio AJ, Segelbacher G, Seddon PJ, Alphey L, Bennett EL, Carlson RH, et al. Is it time for synthetic biodiversity conservation? Trends Ecol Evol. 2017;32:97-107.*

*Barnosky AD, Matzke N, Tomiya S, Wogan GO, Swartz B, Quental TB, et al. Has the Earth’s sixth mass extinction already arrived? Nature. 2011; 471:51-57.*

Speaking of genome editing, this section of the review is currently insufficient. The authors are advised to consult a genome editing specialist to expand upon the existing tools. A comprehensive list of reagents, along with their specific applications, intended gene targets, and desired outcomes, would greatly enhance the manuscript’s utility.

*Our reply: Thank you for your constructive suggestions. Since we have recognized some technical bottlenecks that occurred for threatened plant species, in the revised version we have newly included an important paragraph in the section “The basis for genome editing: Genome resources and hurdles to the implementation of CRISPR tools”. We believe that this new paragraph will mitigate the technical concerns. To make the paragraph more concrete and objective, one of the authors (Prof. Emeritus Myong Gi Chung) consulted Dr. Sang-Tae Kim at the Catholic University of Korea for the discussion of the application of GE in endangered plant species. For clarity, we have also added the “Genome editing tools” and “Efficiency of GE” parts in the newly added Table 1 in which readers can easily get the reagent information required.*

A visual representation, such as a figure or roadmap, outlining the strategic use of gene editing in plant conservation would be highly beneficial. This review should not only inform but also stimulate intellectual curiosity. It should pose challenging questions and suggest innovative solutions, thereby providing readers with a compelling narrative and actionable insights.

*Our reply: Again, thanks for your helpful and constructive suggestions. We have revised Fig. 3 to outline the strategic use of GE in plant conservation by adding “populations crucial to food security and sustainability”, “risk assessment of GE plants”, etc. We have also added some challenging questions (e.g., Under what circumstances is GE technology applied among endangered plants compared with traditional population genetics/genomics and what is the most scientific and efficient way to select target species?; How are deleterious mutations discovered in threatened and endangered plants?; What about the future of GE technology in plant conservation?) and suggested solutions (which are included in the revised text) to activate curiosity and provide possible feasibility.*

**Second round of review**

**Reviewer 1**

The authors have meticulously addressed our concerns and substantially revised the manuscript by elucidating the experimental conditions under which genome editing was conducted, as well as by providing illustrative examples. Nevertheless, the response to the second query has not adequately allayed our apprehensions regarding the potential ecological ramifications of plants that have been subjected to genome editing.

The preservation of the environment and its ecosystems is a matter of profound importance, indispensable to the discourse presented within this article. The employment of genome editing as a means to safeguard plant life represents an innovative methodology, though not without considerable risk. The application of genome editing in the realm of plant conservation diverges substantially from its utilization in agricultural produce. Prior to their introduction into cultivation, genetically edited crops are subjected to exhaustive safety evaluations, and they endure rigorous regulatory oversight throughout their growth cycle. The paramount apprehension we harbor pertains to the unquantified influence that genome-edited flora may exert upon the ecological milieu, rendering assurances of their security elusive.

The incremental portion of the manuscript does not explicitly delineate the potential ecological ramifications of genetically engineered flora under threat, nor does it elucidate concomitant remedial strategies. The instance of Cry3Bb Bt maize cited within the text is inadequate, as genetically modified vegetation may precipitate latent issues such as the exacerbation of weed proliferation, leading to the displacement and even extirpation of indigenous plant species (Bauer-Panskus et al., 2020). In the evaluation of the impact that genetically edited botanicals exert upon the comprehensive ecosystem, it is imperative to encompass a more extensive array of ecological determinants. Although the section "Challenges facing plant conservation using genome editing" mentions "the seven stages of risk assessment" and "cut-off criteria" for risk assessment standards, there is still a lack of specific measures to prevent the negative effects of genome edited plants on the ecological environment. The text in line 423, "GE can make substantial contributions to forest management, and the traits that have shown great promise based on field trials of GE trees are highly diverse, control……. GE trees have tremendous potential as a safe and powerful tool for promoting forest resilience and sustainability," merely accentuates the potential utility of botanical preservation within forest management, yet neglects to stipulate safeguards for its judicious employment.

Hence, I recommend that the authors more thoroughly underscore the possible complications associated with utilizing genome editing in plant preservation and advocate for the implementation of efficacious strategies from the outset, to guarantee the secure application of this technology to imperiled species.

**Reviewer 3**

In the manuscript, the authors proposed very interesting views of genome editing technology on plant conservation. The concept is constructive and is high valuable to response the worldwide ecological challenges. Although the manuscript has been largely improved and is well-written, I still have some major concerns.

1. I have looked through the responses to the former questions. Most of them are reasonable, however, the answer to Q2 of Review 1 was incomplete. The CRISPR introduction of certain genetic variations have potential risks of decreasing biodiversity of plant species.

2. Conventional mutagenesis methods also increase biodiversity for ecology system. It will be more comprehensive to compare the methods to GE.

3. It is recommended to propose a landmark event for plant species recusing, such as the similar example of Mammoth de-extinction.

4. Multiplex genome editing may be much more useful than editing on single locus, which can further discuss.

**Reviewer 4**

After reading the related information carefully, I think the authors added clear description and have addressed the concerns from reviewer 2.

There are a few minor issues that need to be addressed:

1. It is recommended that "gene editing" in Figure 3 be revised to "genome editing" to maintain consistency with the rest of the text.

2. While a relatively detailed description has been provided for the types, components, and editing principles of BE, for the more powerful PE, the author only briefly introduced its composition without explaining its working principle. Therefore, this part could be supplemented.

3. In Box 2, the author mentioned that multiple variants of Cas9 protein have been evolved to recognize different PAMs, but only references to VQR, VRER, and other variants from 2015 are cited. However, the most widely used and powerful variants published in 2020 are SpG and SpRY, so at least these studies should be cited prominently.

4. Box 2 lacks the relevant references mentioned by the author regarding Cas12b and CasX.

**Authors Response**

**Point-by-point responses to the reviewers’ comments:**

Reviewer #1:

The authors have meticulously addressed our concerns and substantially revised the manuscript by elucidating the experimental conditions under which genome editing was conducted, as well as by providing illustrative examples. Nevertheless, the response to the second query has not adequately allayed our apprehensions regarding the potential ecological ramifications of plants that have been subjected to genome editing.

The preservation of the environment and its ecosystems is a matter of profound importance, indispensable to the discourse presented within this article. The employment of genome editing as a means to safeguard plant life represents an innovative methodology, though not without considerable risk. The application of genome editing in the realm of plant conservation diverges substantially from its utilization in agricultural produce. Prior to their introduction into cultivation, genetically edited crops are subjected to exhaustive safety evaluations, and they endure rigorous regulatory oversight throughout their growth cycle. The paramount apprehension we harbor pertains to the unquantified influence that genome-edited flora may exert upon the ecological milieu, rendering assurances of their security elusive.

The incremental portion of the manuscript does not explicitly delineate the potential ecological ramifications of genetically engineered flora under threat, nor does it elucidate concomitant remedial strategies. The instance of Cry3Bb Bt maize cited within the text is inadequate, as genetically modified vegetation may precipitate latent issues such as the exacerbation of weed proliferation, leading to the displacement and even extirpation of indigenous plant species (Bauer-Panskus et al., 2020). In the evaluation of the impact that genetically edited botanicals exert upon the comprehensive ecosystem, it is imperative to encompass a more extensive array of ecological determinants. Although the section “Challenges facing plant conservation using genome editing” mentions “the seven stages of risk assessment” and “cut-off criteria” for risk assessment standards, there is still a lack of specific measures to prevent the negative effects of genome edited plants on the ecological environment. The text in line 423, “GE can make substantial contributions to forest management, and the traits that have shown great promise based on field trials of GE trees are highly diverse, control……. GE trees have tremendous potential as a safe and powerful tool for promoting forest resilience and sustainability,” merely accentuates the potential utility of botanical preservation within forest management, yet neglects to stipulate safeguards for its judicious employment.

Hence, I recommend that the authors more thoroughly underscore the possible complications associated with utilizing genome editing in plant preservation and advocate for the implementation of efficacious strategies from the outset, to guarantee the secure application of this technology to imperiled species.

*Our reply: Thanks for your comprehensive and detailed feedback. We agree with you that GE plants may have potential ecological ramifications. We acknowledge the potential ecological risks associated with GE plants, particularly in conservation contexts distinct from agricultural use. To address this concern comprehensively, we have added the ‘potential ecological ramifications of GE plants and strategies to mitigate negative impacts’ in the revised manuscript below.*

*Like other genetically modified organisms, GE plants for conservation may introduce unexpected risks that harm communities and ecosystems. Additionally, GE plants for conservation are also different from those for agricultural uses, which are more restricted to managed systems. For example, GE plants for agricultural uses that are more product-focused can be planted in a restricted area where they have less communication with wild relatives and thus minimize ecological adverse effects. In contrast, once GE plants for conservation are released to their original habitats or other habitats, it may not be possible to control or recall them. Therefore, we may pay more attention to the ecological ramifications of GE plants for conservation. These dangers include risks to non-target organisms (NTOs) and biodiversity risks, gene flow risks, risks to the evolution of resistance in target species, and weediness risks (Snow and Palma, 1997; Andow and Zwahlen, 2006; Tsatsakis et al., 2011; Bauer-Panskus et al., 2020). NTOs impacted by GE plants include beneficial species, non-target herbivores, soil organisms, species of conservation concern, and species contributing to local biodiversity (Snow et al., 2005). Although most of the NTO studies were based on limited focal species in lab and small-scale field studies, they already showed some negative effects of genetically engineered organisms (GEOs) on NTOs. For example, adverse tritrophic level impacts of transgenic Bt corn pollen and Bt sprays on the green lacewing (Chrysoperla carnea) have been observed in laboratory studies (O'Callaghan et al., 2005). Moreover, Cry1Ab was found to be released into the soil through corn root exudates and can persist for up to nearly one year (Saxena et al., 2002), and Cry1Ab in Bt corn litter can last for at least eight months (Zwahlen et al., 2003), both suggesting potential negative impacts on soil biodiversity and ecosystem functioning. Therefore, we should be aware that the effects of negative NTOs of GE plants may result in the disruption of biotic communities or the loss of species or genetic diversity (Rissler and Mellon, 1996). These consequences might be particularly pronounced in communities that are highly dependent on redundancy of ecological function, or they may have no discernible impact.*

*It is also possible that GE plants can pass their modified gene by gene flow into populations of wild relatives in which the modified gene can be fixed (genetic assimilation). The consequences of this genetic assimilation may reduce the genetic diversity of wild populations and change the fitness of hybrids (Wolfenbarger et al., 2000). Although GE plants may provide new genes or variants that can defeat target pathogens or insects by different mechanisms, conferring higher fitness than their donor plants, the risks of new resistance to target pathogens or insects are still possible. Moreover, even for pathogens that infect multiple host species in a local plant community, the ability to infect a specific host species may change over time in response to fluctuations in the relative abundance of various host species (Parker and Gilbert, 2004). Finally, the persistence or invasiveness of GE plants in their natural habitats or outside their habitats for conservation may induce concern about irreversible eﬀects on biodiversity.*

*Considering these risks, ecological risk assessments (EcoRA) of GE plants are necessary before their release to nature. We advocate for a tiered risk assessment approach (Poppy, 2000), which includes assessment of hazard/exposure (Tier 0), single high dose and dose-response testing (Tier 1), refined hazard characterization and exposure assessment (Tier 2), further refined risk assessment (Tier 3), and additional tier (Tier 4) (Garcia-Alonso et al., 2006). Following this framework, we also recommend EcoRA for GE plants to be conducted case-by-case because the non-target organisms that may be impacted will vary based on the specific GE plant, the trait that has been introduced, and the environment in which the plant is released. In turn, these variabilities complicate the tasks of EcoRA. Recently ecosystem service has been strongly advocated as a key outcome variable in risk assessment to connect ecological conservation and human well-being (Munns et al., 2016).*

*All the above information was summarized and added to lines 398-430 (pp. 18 - 20) under the section “Challenges facing plant conservation using genome editing” and the revised Fig. 3 in the revised manuscript.*

*References:*

*Andow, D. A., & Zwahlen, C. (2006). Assessing environmental risks of transgenic plants. Ecology Letters, 9(2), 196-214.*

*Bauer-Panskus, A., Miyazaki, J., Kawall, K., & Then, C. (2020). Risk assessment of genetically engineered plants that can persist and propagate in the environment. Environmental Sciences Europe, 32(1), 32.*

*Garcia-Alonso, M., Jacobs, E., Raybould, A., Nickson, T. E., Sowig, P., Willekens, H., ... & Tencalla, F. (2006). A tiered system for assessing the risk of genetically modified plants to non-target organisms. Environmental Biosafety Research, 5(2), 57-65.*

*Munns Jr, W. R., Rea, A. W., Suter, G. W., Martin, L., Blake‐Hedges, L., Crk, T., ... & Barron, M. G. (2016). Ecosystem services as assessment endpoints for ecological risk assessment. Integrated environmental assessment and management, 12(3), 522-528.*

*O'Callaghan, M., Glare, T. R., Burgess, E. P., & Malone, L. A. (2005). Effects of plants genetically modified for insect resistance on nontarget organisms. Annual Review of Entomology, 50(1), 271-292.*

*Parker, I. M., & Gilbert, G. S. (2004). The evolutionary ecology of novel plant-pathogen interactions. Annual Review of Ecology, Evolution, and Systematics, 35(1), 675-700.*

*Poppy, G. (2000). GM crops: environmental risks and non-target effects. Trends in Plant Science, 5(1), 4-6.*

*Rissler, J., & Mellon, M. (1996). The Ecological Risks of Endangered Crops. Cambridge, MA: The MIT Press. xii+168 pp.*

*Saxena, D., Flores, S., & Stotzky, G. (2002). Vertical movement in soil of insecticidal Cry1Ab protein from Bacillus thuringiensis. Soil Biology and Biochemistry, 34(1), 111-120.*

*Snow, A. A., & Palma, P. M. (1997). Commercialization of transgenic plants: potential ecological risks. BioScience, 47(2), 86-96.*

*Snow, A. A., Andow, D. A., Gepts, P., Hallerman, E. M., Power, A., Tiedje, J. M., & Wolfenbarger, L. L. (2005). Genetically engineered organisms and the environment: Current status and recommendations 1. Ecological Applications, 15(2), 377-404.*

*Tsatsakis, A. M., Nawaz, M. A., Kouretas, D., Balias, G., Savolainen, K., Tutelyan, V. A., ... & Chung, G. (2017). Environmental impacts of genetically modified plants: a review. Environmental Research, 156, 818-833.*

*Wolfenbarger, L. L., & Phifer, P. R. (2000). The ecological risks and benefits of genetically engineered plants. Science, 290(5499), 2088-2093.*

*Zwahlen, C., Hilbeck, A., Gugerli, P., & Nentwig, W. (2003). Degradation of the Cry1Ab protein within transgenic Bacillus thuringiensis corn tissue in the field. Molecular Ecology, 12(3), 765-775.*

Reviewer #3:

In the manuscript, the authors proposed very interesting views of genome editing technology on plant conservation. The concept is constructive and is high valuable to response the worldwide ecological challenges. Although the manuscript has been largely improved and is well-written, I still have some major concerns.

*Our reply: Thanks for your positive comments.*

1. Conventional mutagenesis methods also increase biodiversity for ecology system. It will be more comprehensive to compare the methods to GE.

*Our reply: Thank you for this suggestion. We acknowledge that conventional mutagenesis, such as chemical mutagens (e.g., ethyl methanesulfonate[EMS]) or physical irradiation (e.g., fast neutrons), can indeed contribute to increasing biodiversity in ecological systems. However, it is important to note that these methods are more random and less targeted than GE, which significantly increases the time and cost involved in selecting suitable individuals for conservation purposes. Typically, mutations induced by conventional mutagenesis tend to be heterozygous. In selfing plants, these mutations can be converted to homozygosity by selfing following Mendelian law. However, this approach is challenging for outcrossing species because of the difficulty in finding two individuals carrying the same mutations for effective breeding. Moreover, the random nature of mutagenesis often leads to a high mutation load, which might reduce the plant fitness (Jung and Jill, 2021). We have incorporated these considerations into our revised manuscript and cited the related reference (lines 103-106, p. 5).*

*Reference:*

*Jung, C., & Till, B. (2021). Mutagenesis and genome editing in crop improvement: perspectives for the global regulatory landscape. Trends in Plant Science, 26(12), 1258-1269.*

2. It is recommended to propose a landmark event for plant species rescuing, such as the similar example of Mammoth de-extinction.

*Our reply: Thanks for your inspiring idea and suggestion. The Mammoth de-extinction project aims to introduce mammoth genes (by using GE) into the Asian Elephant to increase the resilience of habitats to climate change and environmental upheaval. However, unlike animals, plants have endured less impact from the dramatic glacial cycles over the past 2 million years. Instead, they are now facing a unique set of evolutionary challenges, i.e., fire and introduced pests and diseases in the Anthropocene (Canon and Petit, 2020).*

*To date, plant de-extinction has only been achieved by germinating or in vitro tissue-culturing old seeds or spores available in herbarium specimens (Albani Rocchetti et al., 2022). Therefore, there is an urgent need for conservation projects similar to the Mammoth de-extinction but focused on plants, leveraging new technologies like GE (see lines 459-461, p. 21).*

*References:*

*Albani Rocchetti, G., Carta, A., Mondoni, A., Godefroid, S., Davis, C. C., Caneva, G., ... & Abeli, T. (2022). Selecting the best candidates for resurrecting extinct-in-the-wild plants from herbaria. Nature Plants, 8(12), 1385-1393.*

*Cannon, C. H., & Petit, R. J. (2020). The oak syngameon: more than the sum of its parts. New Phytologist, 226(4), 978-983.*

3. Multiplex genome editing may be much more useful than editing on single locus, which can further discuss.

*Our reply: Thanks for your suggestion. Multiplex genome editing offers the advantage of manipulating more sites simultaneously, which significantly reduces the time required to generate variants with multiple advantageous mutations. This capability enhances the adaptive potential of plants. We have included this in our revised manuscript (lines 106-108, p. 5).*

Reviewer #4:

After reading the related information carefully, I think the authors added a clear description and have addressed the concerns from reviewer 2.

There are a few minor issues that need to be addressed:

1. It is recommended that “gene editing” in Figure 3 be revised to “genome editing” to maintain consistency with the rest of the text.

*Our reply: Done.*

2. While a relatively detailed description has been provided for the types, components, and editing principles of BE, for the more powerful PE, the author only briefly introduced its composition without explaining its working principle. Therefore, this part could be supplemented.

*Our reply: Thanks for your comments and suggestions, the working principle of PE (prime editing) was added to Box 2 (lines 620-633, pp. 28 - 29) in the revised manuscript.*

3. In Box 2, the author mentioned that multiple variants of Cas9 protein have been evolved to recognize different PAMs, but only references to VQR, VRER, and other variants from 2015 are cited. However, the most widely used and powerful variants published in 2020 are SpG and SpRY, so at least these studies should be cited prominently.

*Our reply: Thanks for your suggestions, SpG and SpRY studies were cited in Box 2 (line 582, p. 26).*

4. Box 2 lacks the relevant references mentioned by the author regarding Cas12b and CasX.

*Our reply: Thanks for your suggestion, relevant references were added to Box 2 (lines 586, and 589, p. 27).*

**Third round of review**

**Reviewer 1**

In this revision, the authors have provided the explanation of the potential ecological impacts that could arise from utilizing genome editing for plant protection. Acknowledging these risks, the authors include enhanced sections on eco-risk assessments (EcoRA) of genetically engineered (GE) plants, with the aim to minimize such risks and endeavor to safely apply genome editing technology in rescuing threatened or endangered plant species. However, this should be viewed as a compromise approach; it is advisable not to employ this method unless absolutely necessary, which must be emphasized again within the text, especially in the abstract. I have no further question of this manuscript.

**Reviewer 3**

I have no further comment.

**Authors Response**

**Point-by-point responses to the reviewers’ comments:**

Reviewer #1:

In this revision, the authors have provided the explanation of the potential ecological impacts that could arise from utilizing genome editing for plant protection. Acknowledging these risks, the authors include enhanced sections on eco-risk assessments (EcoRA) of genetically engineered (GE) plants, with the aim to minimize such risks and endeavor to safely apply genome editing technology in rescuing threatened or endangered plant species. However, this should be viewed as a compromise approach; it is advisable not to employ this method unless absolutely necessary, which must be emphasized again within the text, especially in the abstract. I have no further question of this manuscript.

*Our reply: Thank you for your suggestion, we have revised the Abstract accordingly.*
